# Recent Advances in the Fabrication of Structural Nanocellulose‐Loaded Functionalized Biochar‐Based Biopolymeric Nanocomposites for Industrial Wastewater Treatment by Continuous Adsorption With Modeling: A Cutting‐Edge Review

**DOI:** 10.1002/gch2.70107

**Published:** 2026-04-24

**Authors:** Md. Mahmudur Rahman, M Mohinur Rahman Rabby, G.M Musfiq Ismam, Md. Khalid Al Zuhanee, Salah Knani, Reem Alreshidi, Faisal Ahmed Naiem

**Affiliations:** ^1^ BCSIR Rajshahi Laboratory Bangladesh Council of Scientific and Industrial Research (BCSIR) Rajshahi Bangladesh; ^2^ Department of Chemical Engineering Rajshahi University of Engineering and Technology (RUET) Rajshahi Bangladesh; ^3^ Department of Mechanical Engineering Rajshahi University of Engineering and Technology (RUET) Rajshahi Bangladesh; ^4^ Center for Scientific Research and Entrepreneurship Northern Border University Arar Saudi Arabia; ^5^ Department of Physics College of Science Northern Border University Arar Saudi Arabia

**Keywords:** agro‐waste biomass‐derived CNC and Functionalized biochar (FBC), CNC‐FBC biopolymeric nanosorbents, fixed‐bed column continuous adsorption, mathematical models and experimental BTC curve, state‐of‐the‐art fabrication methods, synergistic adsorption mechanism

## Abstract

Production of multifunctional bionanoadsorbents from agrowaste/plant biomass‐derived CNC/FBC by developing an economic/environmentally friendly technology for bulk‐scale wastewater treatment is a critical issue. Every moment, a massive amount of hazardous effluent is expelled from several large/medium/small‐scale industries directly into the environment, which can destroy the ecological balance/public security. Hence, in this overview, several advanced production techniques have been proposed addressing the effective fabrication of CNC‐FBC‐based bionanoadsorbents that could be beneficial in wastewater treatment for their outstanding properties, structural purity, and upgradation. Moreover, numerous potential characterization techniques, beneficial mode of application, and synergistic mechanism including chemical adsorption due to strong interaction, both monolayer/multi‐layer physical adsorption due to weak interaction, interparticular diffusion/deep penetration due to higher surface area, mesoporous microstructure with pore tube/canal of the considered bionanocomposites have been suggested for a better understanding about the scope, significance, applicability, and selectivity. However, the CNC‐FBC bionanocomposites could be more selective and beneficial than others because of their low cost, higher efficiency, greater stability, structural purity, highly dense binding sites, negative surface charge, and reusability. This review also offers the modification techniques and mathematical modeling for enhanced prediction of the experimental BTC curve and actual route adsorption by detecting the reaction kinetics/thermodynamics/isotherms and energy interpretation.

## Graphical abstract

This study focused on the advanced fabrication and application of agro‐waste biomass/natural lignocellulosic fiber‐derived structural CNC‐FBC‐based multifunctional biopolymeric nanoadsorbents in industrial wastewater treatment technology. These CNC‐FBC‐based biopolymeric nanosorbents would be a sustainable alternative to fossil‐based, hazardous ones for environmental protection and would facilitate a zero‐waste strategy. The mechanism and modeling could be beneficial for improved prediction and data interpretation.

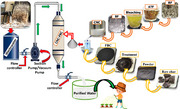

## 1 Introduction

Water is the essential part for all living organisms and a basic need for the growth of life in the environment, cause it has a heavy impact. On the other hand, water pollution has become a common problem in many countries around the world, influencing the freshwater availability globally. Major water bodies include ponds, rivers, lakes, and oceans, which are being polluted promptly. Water pollution shows a depletive concern for the global ecosystem, human health, aquatic life, and also from a socio‐economic perspective [1, 2]. Water is being polluted from different sources sewage leakage, oil spills, mining, herbicides and fertilizers, toxic waste, textile effluent, etc. [3]. Pollutants from these sources can be classified into inorganic pollutants, organic pollutants, radioactive pollutants, etc., which makes water unsafe [4]. Although industrial operations are essential to economic expansion, they also generate significant amounts of wastewater. Disposal of industrial wastewater into the water bodies is a threat to humans and the environment [5]. Presence of heavy metals, organic dyes, and persistent organic compounds in industrial wastewater is a concerning issue because they can disrupt biological functions and accumulate in the food chain [6]. Heavy metals such as lead (Pb), chromium (Cr), cadmium (Cd), mercury (Hg) are non‐biodegradable in nature and retain long‐term toxicity. Lead can cause brain damage and developmental problems, hexavalent chromium can lead to lung problems, mercury causes severe damage to the kidneys, and cadmium is carcinogenic [7]. Environmentally, these heavy metals contaminated water reduces agricultural productivity, harms aquatic life, and enters the food chain through bioaccumulation, posing long‐term ecological threats and health hazards to wildlife and humans alike [8]. However, for better clarity about the real scenario of the industrial wastewater, its bad impact on the aquatic life cycle, other wildlife, public health, and ecosystem, including soil pollution, a sketch diagram has been shown in Figure 1a. Despite advancements in wastewater treatment technologies such as chemical precipitation, ion exchange, membrane filtration, electrochemical methods, etc., there are still limitations in the case of pollutant removal capacity, operational cost, sludge generation, ineffectiveness in the case of particular metal ions, space requirements, process complexity perspective [9]. Therefore, sustainable, cost‐effective industrial water treatment is crucial to reduce pollution and ensure environmental safety. Adsorption is an emerging effective technique for the removal of pollutants from industrial wastewater. Adsorption is a surface‐based process where heavy metal from a liquid accumulates on the surface of a solid material, forming a concentrated layer due to physical or chemical interactions without penetrating into the solid's bulk [10]. It is very effective for removing heavy metals from water because it is selective, affordable, and works even at low concentrations [11]. Traditional adsorbents like activated carbon, bio‐adsorbents, and special polymers can trap heavy metals efficiently, making the process eco‐friendly, reusable for sustainable water treatment [12]. Nevertheless, for a better understanding of the scope, involvement, implication, selectivity, novelty, acceptability, and applicability of this current study and related topics, a diagram has been shown in Figure 1b, indicating the keywords co‐occurrence as per Vosviewer software and Scopus database. The primary drawback of using adsorption to remove heavy metals is that the adsorbent materials may eventually get saturated and need to be replaced or regenerated, which can be expensive and produce secondary waste [13]. Furthermore, temperature, pH, and the existence of competing ions in the water can all have an impact on adsorption efficiency. Implementation of different adsorbents on an industrial scale is challenging because regeneration plays a pivotal role as operational costs are affected [14]. Advanced adsorbents, particularly nano‐material‐based and functionalized bio‐based materials, can be an alternative while exhibiting enhanced adsorption properties, possessing a high surface area, and strong attraction toward target pollutants. Functionalized biochar is a low‐cost and eco‐friendly offering with superior adsorption capability and reusability compared to conventional materials. By incorporating cellulose nanocrystalline (CNC), the adsorption efficiency and selectivity of biochar can be further improved. Biochar is produced by the pyrolysis of biomass inside a closed reactor at temperatures ranging from 300°C to 600°C under limited oxygen conditions. As a result, thermochemical conversion also exhibits a porous structure with high carbon content and the ability to adsorb a wide range of pollutants. One of the key advantages of biochar is its environmental sustainability, as it is derived from agricultural and forestry waste. As a result, dependency on non‐renewable sources also subsides. Additionally, its surface functional groups, such as hydroxyl (─OH), carboxyl (─COOH), and carbonyl (─C═O), play a crucial role in pollutant binding; however, raw biochar often lacks the adsorption efficiency required for industrial‐scale applications [15]. To overcome these limitations, biochar is modified or functionalized to stimulate adsorption capacity. Common chemical modification techniques include acid or base treatments, metal impregnation, biofilm, and nanomaterial loading significantly improve biochar's surface characteristics. Cellulose nanocrystals (CNCs) are isolated from natural sources such as wood or cotton through a process of acid hydrolysis, which targets the breakdown of amorphous cellulose, leaving behind crystalline nanostructures. This extraction method involves multiple steps: pretreatment to remove impurities, acid hydrolysis to degrade non‐crystalline areas, followed by extensive washing, sonication for uniform dispersion, and drying [16]. Due to their high surface area, mechanical robustness, and ability to be functionalized, CNCs are increasingly used in various fields, including environmental applications. Incorporation of cellulose nanocrystal (CNC) into biochar, introduced as CNC‐functionalized biochar, improves pollutant removal efficiency, ensuring better pollutant accessibility. Moreover, CNC‐functionalized biochar and uniform adsorption are suitable for large‐scale industrial wastewater treatment.

FIGURE 1(a) The real scenario of the industrial wastewater, its bad impact on the aquatic life cycle, other wildlife, public health, and ecosystem, including soil pollution, and (b) Co‐occurrence of keywords (such as CNC‐AC bionanocomposites, adsorptions, hazardous pollutants, wastewater treatment, etc.) as per Scopus database from 2015–2025, by applying Vosviewer software.

**Figure:**
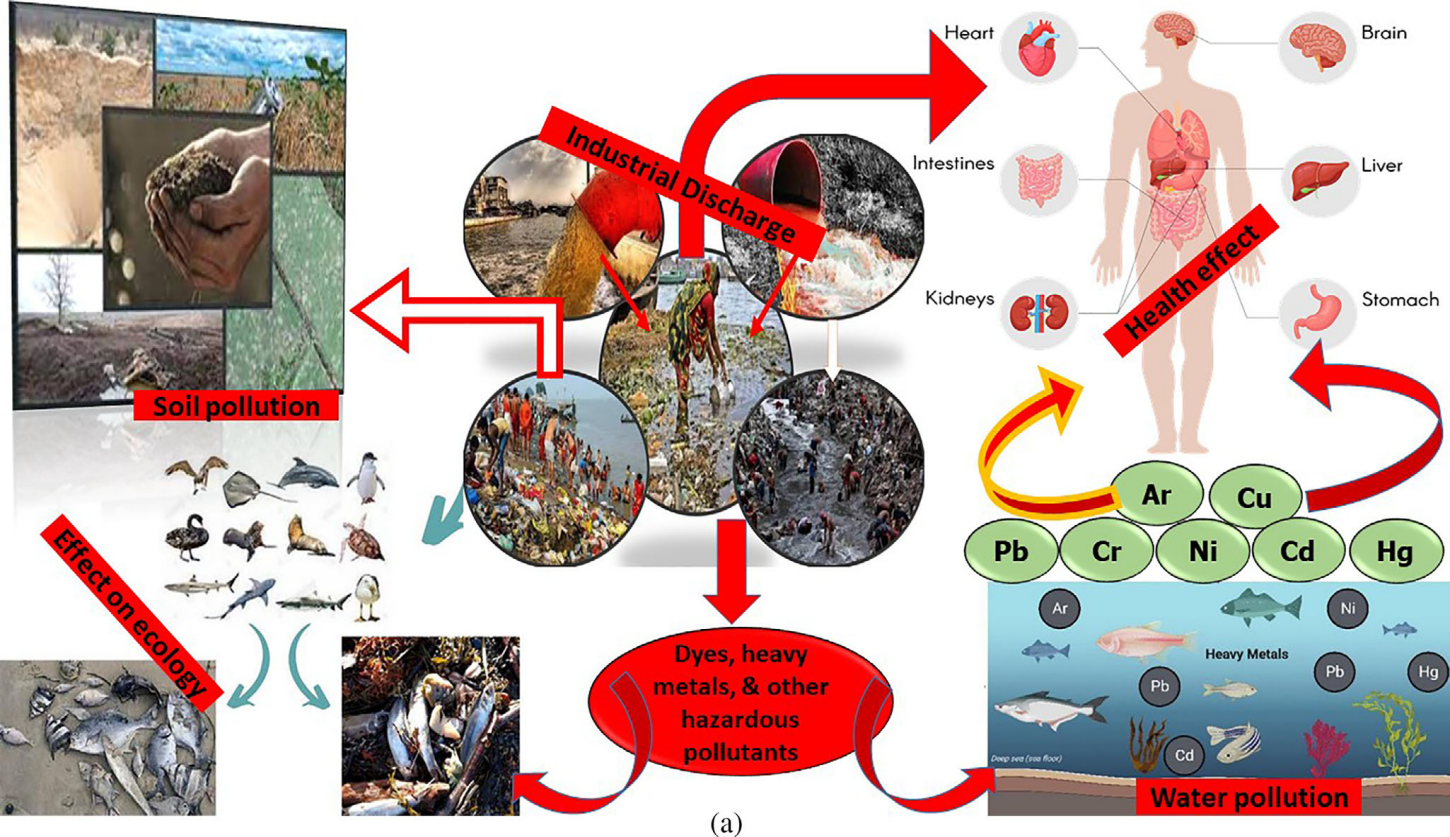

**Figure:**
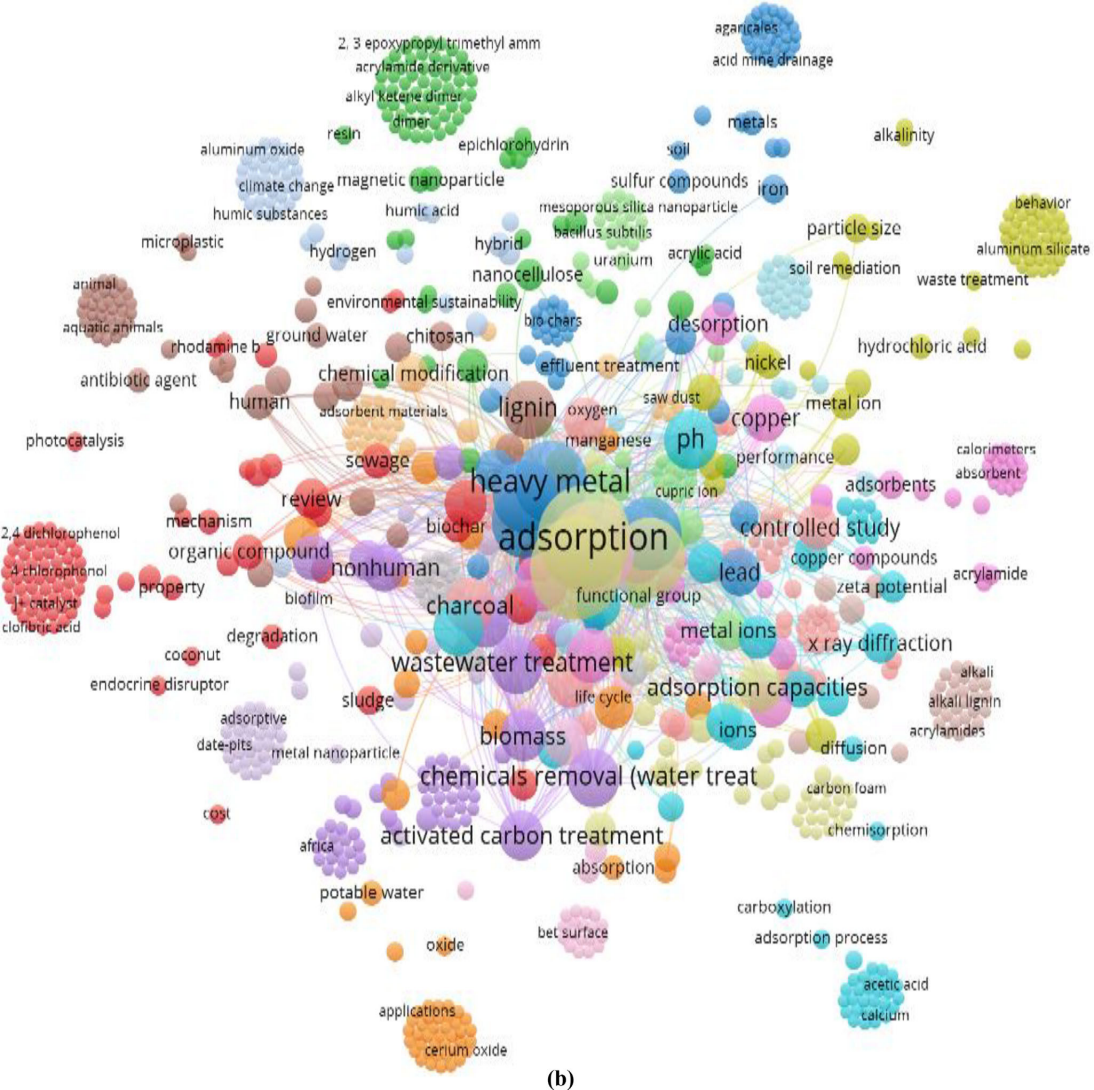

However, despite CNC‐functionalized biochar nanoadsorbents having attracted growing interest for their potential in industrial wastewater treatment, their real‐time and large‐scale application has to be explored. While several studies have investigated the adsorption performance of these materials under controlled laboratory conditions, the translation of this research into practical systems such as continuous‐flow columns has been limited. The fabrication of such materials typically involves the functionalization of biochar with a reactive group, followed by the immobilization of cellulose nanocrystals through covalent bonding. This combination creates a synergistic platform that enhances pollutant uptake via chemisorption, mono‐ and multilayer physisorption, and interparticular diffusion. And previously, no research has focused on combining these mechanisms altogether. However, there is still a lack of comprehensive understanding of how CNCs interact at the molecular level with modified biochar surfaces, especially in complex industrial matrices containing diverse contaminants. Furthermore, though mathematical models have been applied to describe adsorption. There is also limited research on reusability, regeneration efficiency, and structural stability under repeated adsorption‐desorption cycles. Moving forward, future research should focus on standardizing fabrication processes, evaluating long‐term performance under real effluent conditions, and integrating these nanoadsorbents into complex treatment systems. These steps are essential for advancing CNC‐functionalized biochar from promising lab‐scale materials to reliable solutions for industrial wastewater remediation.

## 2 Synthesis of CNC From Natural Plant Fibers

### 2.1 Discussion and Distribution of Cellulose in Plants

**FIGURE 2:**
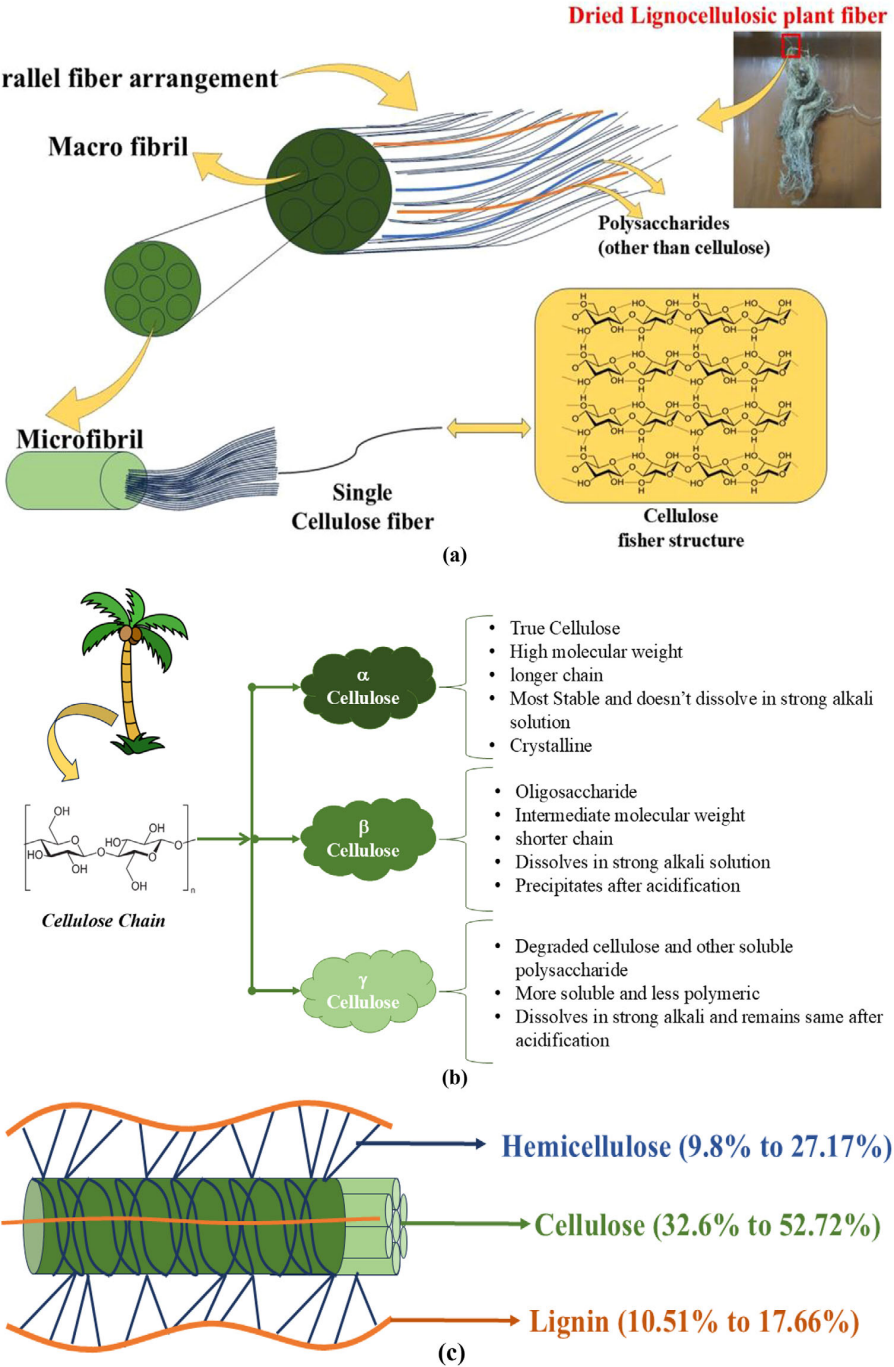
(a) The structural hierarchy of cellulose, indicating its arrangement in naturally available plant fiber, (b) Components and classification of naturally occurring plant cellulose, and (c) Distribution of Hemicellulose, Cellulose, and Lignin in Lignocellulosic Plant fiber by percentage.

Being the most abundant natural and renewable polymer, cellulose is the most important structural component among all three main components of lignocellulose (Hemicellulose, Lignin, and Cellulose) in plants [17, 18, 19]. Along with being integral components of plants, many living organisms like bacteria, fungi, algae, and many sea animals and vegetations have cellulose in their cells and organisms [20]. More than 1.49 × 10^12^ tons of cellulose are produced and used worldwide in a year [21]. Cellulose is an unbranched natural polysaccharide (a class of carbohydrate with 10++ sugar molecules) which is largely found in plants. It is a major part of the cell walls of plant cells. Its role is not to store energy like amylose (St. chain of glucose polymers) or amylopectin (a branched glucose polymer, also a starch that is found in plants) or glycogen (a starch found in animals, which is a highly branched glucose polymer), but rather it serves for structural support. By 1,4‐glycosydic bonds, they form very long cellulose chains which group together by H‐bonds forming microfibrils. Microfibrils also by grouping form microfibrils, which, finally, by grouping form very strong fibers (Shown in Figure 2a). They are generated in plant cells by photochemical biosynthesis. Being a premier integrant of the plant cell membrane and a fundamental constituent of the plant cell, the percentage of cellulose varies widely in various plant species. For instance, it's present in the highest quantities in textile plants (85%–99%) and lowest in some legumes (7%–10%). In other scenarios, for example, in the case of cereal‐type plants, it's 40%–50%, and in the wood mass of different tree species 40%–60%. From a chemical viewpoint, cellulose does not indicate a unitary substance. It's a result of condensation of a variable number of D‐glucose by removing water contained in monosaccharide molecules and is represented by (C_6_H_12_O_6_)n, where n varies from 700–800 and 2500–3000 or even more [22]. In the 1900's, cellulose was described as a combination of 3 types of polysaccharides by Cross and Bevan. These were Alpha Cellulose, Beta Cellulose, and Gamma Cellulose based on sodium hydroxide treatment of the raw cellulose sample [23]. Crystalline cellulose is insoluble in most solvents due to the H‐bonds in regions of crystallinity where hydroxyl radicals are grouped orderly, preventing cellulose from melting. In a non‐crystalline region where the hydroxyl radicals are not ordered, hydroxyl radicals combine with other molecules, resulting in dissolution. This dissolution may be done by means of acidic or basic reagents (calcium bisulfite Ca(HSO_3_)_2_ in the bisulfite process, or a mixture of sodium sulfate NaSO_3,_ and strong sodium hydroxide, NaOH, in the sulfite process) releasing most of the β‐cellulose and γ‐cellulose [short chain amorphous cellulose or monosaccharide that haven't formed cellulose yet] [24]. These three types of cellulose are briefly explained in the following diagram (Figure 2b).

### 2.2 Production of CNC From the Lignocellulosic Plant Fibers

**FIGURE 3:**
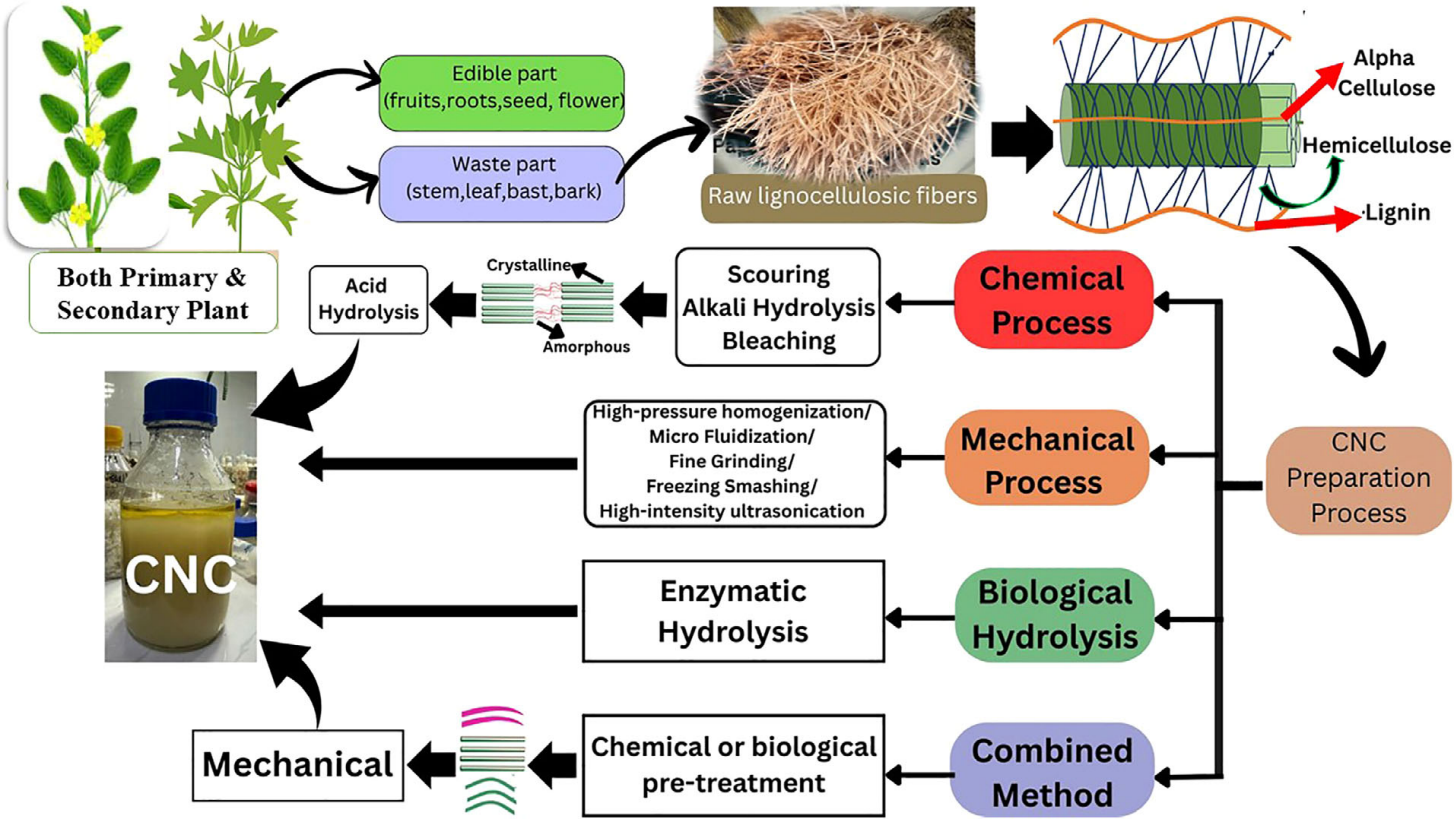
Schematic flow diagram addressing the production route of CNC from several lignocellulosic biomass, including both primary and secondary plants, by following various physical, chemical, mechanical, biological, and physicochemical methods. Adopted from [30] with permission.

Lignocellulose is a structural plant component which the most abundant raw material on the planet, and being a biomass, it can also be treated to become a biofuel [25]. It can be separated into its 3 components: Hemicellulose (9.8% to 27.17%), Cellulose (32.6% to 52.72%) [26, 27] & Lignin (10.51% to 17.66%) [28, 29]. The components of lignocellulose are illustrated clearly (in Figure 2c) below. All these three components can be separated by different physical, chemical, and physicochemical processes [25]. Cellulose itself is very useful in many real‐world applications of pharmaceuticals, medical science, industrial sectors, etc. But being a polymeric material with incomplete crystallization meaning being a combination of both crystalline and amorphous structure, it lacks multifunctional use due to its weakness against water, oxygen, ultraviolet, and corrosive solvents (acidic or basic), along with a lack of anti‐bacterial properties. Development of high‐performance crystalline nanocellulose can help solve this pressing issue. However, these three components of lignocellulose are tightly bound together, forming very strong, complex, and rigid structures [7]. Extracting crystalline nanocellulose (CNC) from this raw biomass (lignocellulose) requires pretreatment involving multiple steps of physical, biological, mechanical, chemical, biochemical, and physicochemical processes [1]. They are shown in Figure 3 and discussed below:

#### 2.2.1 Isolation of Raw Fiber From Plant Biomass

The first and foremost task for CNC fabrication is the extraction of natural lignocellulose fiber. This can be achieved from bacterial decomposition by using methods like dew retting, water retting, or mechanical separation with chemical extraction [1, 31]. Retting processes (water rating, dew rating) are the most common and widely used structure degradation methods. 2–4 weeks of retting in an aqueous environment degrade and dissolve the wax, fat, and adhesive material from the considered fibrous plant portion/biomass from which lignocellulosic fibers are desired, but the time requirement varies depending on the type and category of fibrous plant material [32]. What happens in a retting process is that the aqueous beneficial bacteria break down large cellular tissues into small individual lignocellulose fibers, which are swelled and packed [33, 34]. Apart from this, retting for more time than needed might reduce the mechanical properties of individual fibers and weaken them for further treatment for CNC fabrication [35, 36]. Even after applying proper environmental settings and meeting necessary time requirements, fibers obtained by the retting process are of low quality, which makes it less commendable at the industrial level. For such drawbacks, the mechanical extraction process followed by chemical pretreatment is used. Mechanical extraction involves the use of a decorticator and/or hammer mill to crush dried, chopped cellular tissues containing wax, fat, adhesive portion, and later chemical treatment using N‐Hexane, Ethyl Acetate, Methanol, and distilled water for 3–5 h in Soxhlet apparatus for the removal of wax, fat, pectin, and adhesive materials [35, 37, 38, 39]. Mechanical extraction comes with benefits that are in line with industrial usage and are attested with some disadvantages like higher cost and energy requirements.

#### 2.2.2 Surface Modification Treatment of Raw Fiber

For removing adhered impurities (dust, dirt, fat, wax, etc.), the hydrophilic nature of extracted raw fiber, and its surface modification are crucial for CNC production. Pretreatment (such as scouring, alkali treatment, and bleaching operation, etc.) helps to disrupt the lignocellulose structure by delignification and makes cellulose more accessible for the production of more pure CNCs [5, 39]. However, for better clarity, these processes have been described below:

(a) Scouring is done as the preliminary stage of the pretreatment process, which serves to enhance the bleaching effectiveness of the raw fiber. This can be conducted by surface‐active agents like detergents or soda. Rahman and co‐workers used a 10% laundry soap solution with a 50:1 soap solution to fiber ratio, keeping it for 30 min at 60 degrees. Successful scouring can be ensured by the physical appearance of the fiber [34]. Removing dust, dirt, lignin, pectin, and any fatty or gummy substance that was left after bacterial decomposition or chemo‐mechanical treatment in the cuticle layer is the main target of scouring, which otherwise could reduce the efficiency of CNC production by restricting contact with alkali and other acidic solvents. (b) After scouring, alkali treatment is done by immersing treated fiber into a strong alkali solution (NaOH or KOH) to expose the smaller crystalline structure of cellulose by dissolving a specific portion of hemicellulose and other impurities (other substances than cellulose) that coat the outer cell wall surface [40, 41, 42]. Alkali treatment disrupts the hydroxyl bonding within the fiber network which results in alkoxide production [7, 43]. For better clarity, a general equation has been shown by following the reaction.

(1) $$\begin{array}{ccc} & & \mathrm{NaOH}/\mathrm{KOH}+\mathrm{Fiber}\;-\;\mathrm{OH}\;\\ & & \to \;\mathrm{Fiber}\;-\;O-\;\mathrm{Na}+(\mathrm{Alkoxide})+H2O \end{array}$$

Producing significant alteration, this pretreatment dissolves the cementing components (hemicellulose and lignin) of lignocellulose structure after separating interfibrillar areas from fibers, which breaks down to the separation of cellulose fibril bundles [40, 42, 44]. Strong base‐induced hydrolysis dissolves densely branched hemicellulose and depolymerizes lignin [42, 45]. Mahafujul Hassan and his co‐workers employed a 17% NaOH solution at 80 degrees for 4 h at a 1:18 fiber to solution ratio to separate alpha cellulose from lignin and hemicellulose matrix [46]. (c) After the alpha cellulose is filtered from most of the lignin and hemicellulose portion, it is bleached to remove even the smallest residue of lignin or hemicellulose present because they impede the extrication of CNC, especially lignin, as they are most difficult to get rid of by alkali treatment [14, 40, 47]. Bleaching is the most effective step of lignocellulose pretreatment for the removal of lignin. Hence, simply termed as delignification [48, 49]. In the bleaching process alkali alkali‐treated fibers are boiled with sodium chlorite (NaClO_2_) solution in an acidic environment [32, 50]. Chlorine dioxide is released from sodium chloride (in an acidic environment, chlorine is not produced; instead, chlorine oxide is produced [51]. Oxidizes lignin residue by attacking the aromatic ring structure of lignin, consequently performing more fibrillation of alkali‐treated fiber. Also, hemicellulose, pectin (if any present), undergoes oxidation, resulting in further opening of fiber bundles [34, 43, 52]. After proper bleaching, the acidic oxidizing environment must be neutralized via a reducing agent. It would help to neutralize any bleaching agent that might be present in the fiber after bleaching. 2% sodium metabisulfite (Na_2_S_2_O3) solution might be used as the reducing environment. After this, the fibers must be rinsed with distilled water, followed by proper drying. The dried fibers might be stored in a desiccator and termed as ‘single bleached fiber’ [46]. It also increases the surface area and porosity of cellulose, which makes it more accessible to enzymes and chemicals used in hydrolysis for CNC production [53].

#### 2.2.3 Production of CNC From Bleached Fiber

Cellulose fibrils extracted after bleaching have a crystalline structure that consists of closely packed nanocrystals with hydrogen bonding between them. These bonds are disordered and amorphous with imperfect axial orientation, which compromises the noble properties of CNC [54, 55]. These deleterious amorphous non‐crystalline regions must be removed to isolate highly crystalline purified CNC. Nanocellulose might be prepared by using a top‐down approach or a bottom‐up approach. Top‐down involves using plant sources (primary or secondary) and by various chemo‐mechanical processes convert them to CNC and the bottom‐up approach uses smaller molecules like monosaccharides or hydrolyzed sugar to synthesize CNC in a culture medium via bacterial and microbial culture. More or less, most of the methods of CNC fabrication involve two main stages: Cellulose extraction by multiple pretreatment mechanisms (Hemicellulose, Lignin, and Pectin are removed) and CNC extraction by acid or ionic hydrolysis processes (amorphous regions are removed). In acid hydrolysis for CNC fabrication, the most common acids are strong mineral acids (such as Sulfuric acid, Hydrochloric acid, Nitric acid, etc.), organic acids (like Oxalic acid, Citric acid, and Formic acid), and solid acids (like Phospho‐tungstic acid) [46, 49, 56, 57]. Compared to mineral acid, organic and solid acids have less impact on equipment, and residue recycling is comparatively easy, but it has the drawback of a lower CNC production rate and efficiency, and sometimes require additional treatments. Basically, acids liberate hydrogen (H+) or hydronium ions (H_3_O^+^) that break down the glycosidic linkages of amorphous cellulose fibrils. So, the hierarchical structure of the nano fibrils breaks down and initiates the formation of CNC [49, 57, 58, 59]. According to the recent report of Hassan and co‐workers, after acid hydrolysis for 30–40 min, the quenched (to 5–10‐fold with excess of cold water) and cooled mixture is centrifuged and rinsed to achieve neutral pH [60, 61, 62, 63]. Then, to make a suitable colloidal dispersion, the nano‐cellulose extracted is preserved with an appropriate amount of ethanol [46, 64].

### 2.3 Structure, Properties, and Application of CNC

Nano‐cellulose is a high‐performance engineering material obtained from bioresources. They are environmentally friendly in nature and have interesting physical (biodegradability, low thermal expansion, optical transparency [65, 66], chemical and mechanical properties [1]. Due to such promising properties, nanocellulose is widely used in many fields. The dimension of cellulose nanocrystals depends on the nature of the source material. The width and length depend on time, temperature, and purity of the source of cellulose [67, 68]. The following table (Table 1) shows the dimensions of nanocellulose obtained from various sources.

**TABLE 1:** Different dimensions, including the length, width, and aspect ratio of the extracted CNC by conducting acid hydrolysis methods and produced from various naturally occurring lignocellulosic plant fibers.

Hydrolysis method	Acid type	Cellulose source	Acid conc. %Wt.	Time (min)	Temp. °C	Length, width, and aspect ratio (nm) of the extracted CNC	Refs.
Sulfuric acid (H_2_SO_4_) hydrolysis	Inorganic	Ramie	64	240	45	L = 70–200; D = 5–15; Aspect ratio (L/D) = ∼12	[69]
Sulfuric acid hydrolysis	Inorganic	Wood	64	25–45	45	L = 100∼300; D = 3∼5; Aspect ratio (L/D) = 20–100	[70]
Sulfuric acid hydrolysis	Inorganic	Sisal	60	30	45	L = 100–300; D = 3∼5; Aspect ratio (L/D) = ∼60	[71]
Sulfuric acid hydrolysis	Inorganic	Tunicates	98	90	60	L = ∼1000; D = 10–20; Aspect ratio (L/D) = ∼100.	[72]
Sulfuric acid hydrolysis	Inorganic	Cotton	65	45	45	L = 100–300; D = 3–5; Aspect ratio (L/D) = 20∼100.	[73]
Oxalic acid (COOH)_2_ hydrolysis	Organic	Eucalyptus Pulp	50–70	45–90	100	L = 273–377; D = ∼15; Aspect ratio (L/D) = 13.4–27.3	[74]
Maleic acid Hydrolysis (HO_2_CCH = CHCO_2_H)	Organic	Bamboo fibers	75	180	110	L = 150; D = 15; Aspect ratio (L/D) = 10	[75]

Nanocellulose can be categorized into three major groups based on source, size, and morphology. They are: i) CNC (cellulose nano crystal), ii) CNF (cellulose nanofibril), iii) BNC (bacterial nano cellulose) [76]. CNC is the result of acid hydrolysis of plant cellulosic material. They have a morphology of whiskers and can be resembled as rod or needle shapes. CNC obtained by acid hydrolysis has an average diameter in the range of 5–70 nm and an average length of 100–250 nm [32, 77, 78]. CNFs are fabricated via strong mechanical disintegration treatment [79] and BNCs via bacteria (cultured) in a carbon source containing nutrient medium [76]. CNC production has been attracting researchers all over the world since the early 2000 s for its unique characteristics. Especially due to the unique physical and chemical properties. The physical properties of CNC can be discussed under four main categories, such as mechanical, rheological, optical, and thermal. Quantitative Measurement of the mechanical properties of nanomaterials are of tremendous challenge due to anisotropy, defects, crystalline percentage, sample dimension, and many other factors that impact in multiple axial directions, affecting results. These are theoretically found by inelastic X‐ray scattering, X‐ray diffraction, Raman Scattering, and AFM (atomic force microscopy) [66]. The tensile strength of CNC found in this manner was 2–7.7 GPa, i.e, sometimes even stronger than the strongest metal wire and Kevlar‐49 [66, 80]. Many Studies found Young's Elastic modulus of CNC near 50–150 GPa and transverse modulus of individual CNC near 18‐50 GPa [81, 82] using AFM with 3D finite element analysis. This can be summarized as superior mechanical strength with low weight and high stiffness. CNC has a crystallinity index of 50%–90% [83]. CNC also possesses good thermal stability, optical properties (transparency), and a wide surface area. CNC has a low thermal expansion coefficient (0.01 ppm/K) and high surface area (100–150 m^2^/g) [84, 85]. But it loses its thermal stability and crystallinity with increasing time duration of acid hydrolysis. Thermal stability and crystallization also depend on cellulose source, processing method, and sulfate content of CNC [31, 83]. Dilute CNC suspensions show shear thinning behavior, i.e., fluid viscosity decreases as shear stress increases. When the shear rate is small, shear thinning depends on the concentration of the suspension of CNC. At higher concentrations, shear thinning is low. Because at higher concentrations, the suspension becomes lyotropic (suspension becomes liquid crystal) and shows anomalous behavior [66]. This phenomenon arises because CNCs, having a rod shape, align themselves and clump together after a critical shear value. The type of acid hydrolysis also affects the rheological behavior of CNC. Studies suggest that hydrochloric acid hydrolysis‐derived CNC show higher shear thinning, and at lower concentration, their viscosity increases with increasing shear stress (anti‐thixotropy), and at higher concentration, their viscosity decreases with time at constant shear value (thixotropy), which is a very unusual behavior of CNC. CNCs are also biocompatible, biodegradable, and renewable (inexhaustible) [86]. The spectacular characteristics of cellulose make them applicable for various applications in several fields such as industrial and domestic wastewater treatment via adsorption, bioplastic manufacturing, drugs carrier in biomedical sector, surgical apparatus production and processing, additionally many engineering, industrial and bulky devises production or preparation as a replacement of the fossil‐based hazardous synthetic materials for sustainable environmental management and monitoring [5, 7, 8, 9, 87]. Some of the most prominent applications of CNC are mentioned (in Figure 4e) for better clarity.

FIGURE 4(a) An overview of the biomass to biochar conversion techniques, (b) Thermo‐chemical conversion techniques of biomass into biochar, (c) An overview of types of the Pyrolysis process with respective biochar yields, (d) An overview of the Gasification process for biochar production, and (e) Some of the most prominent applications of CNCs as a precursor addressing multifunctional biopolymeric nanocomposites manufacturing indicating their importance in human life.

**Figure:**
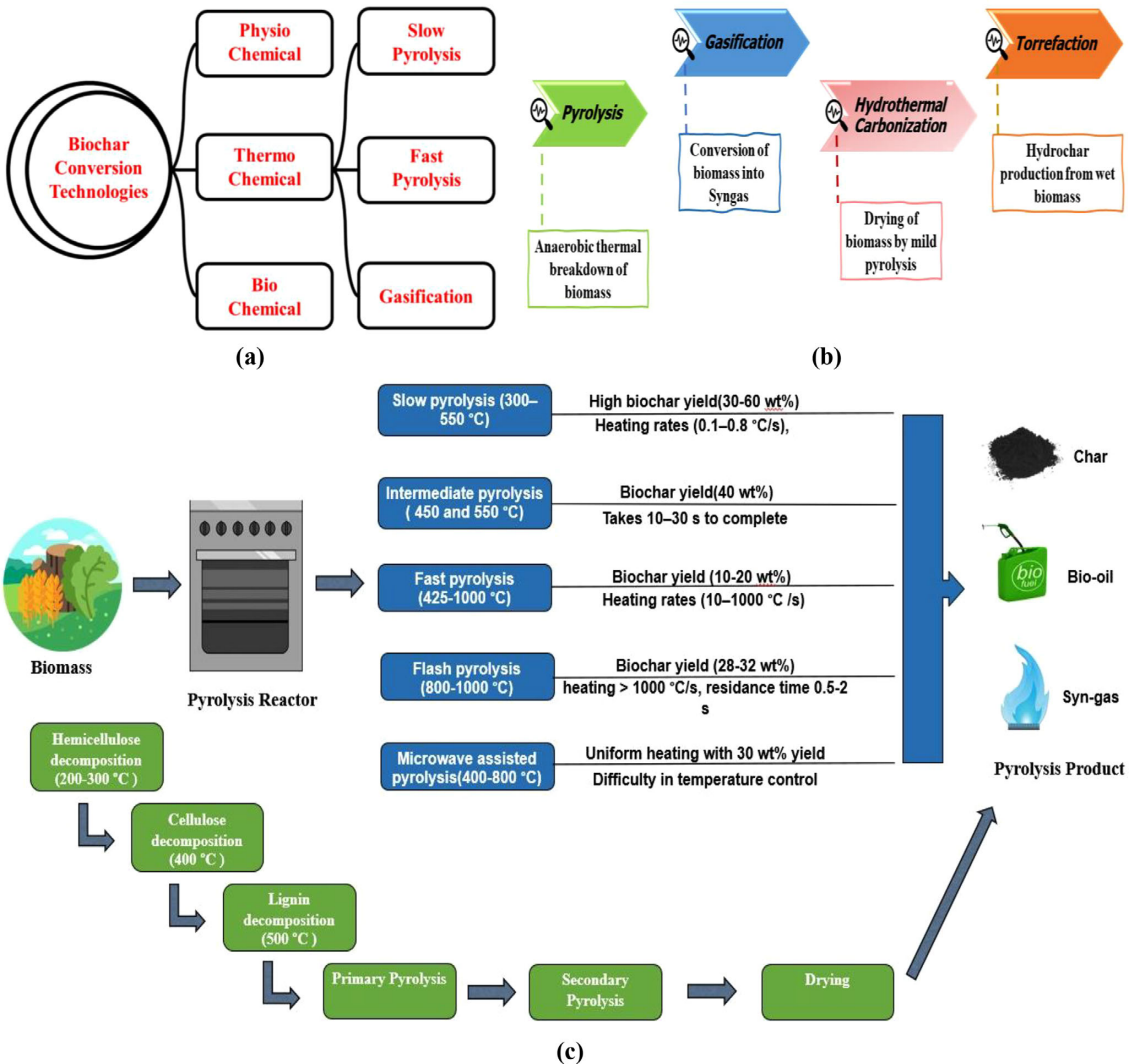

**Figure:**
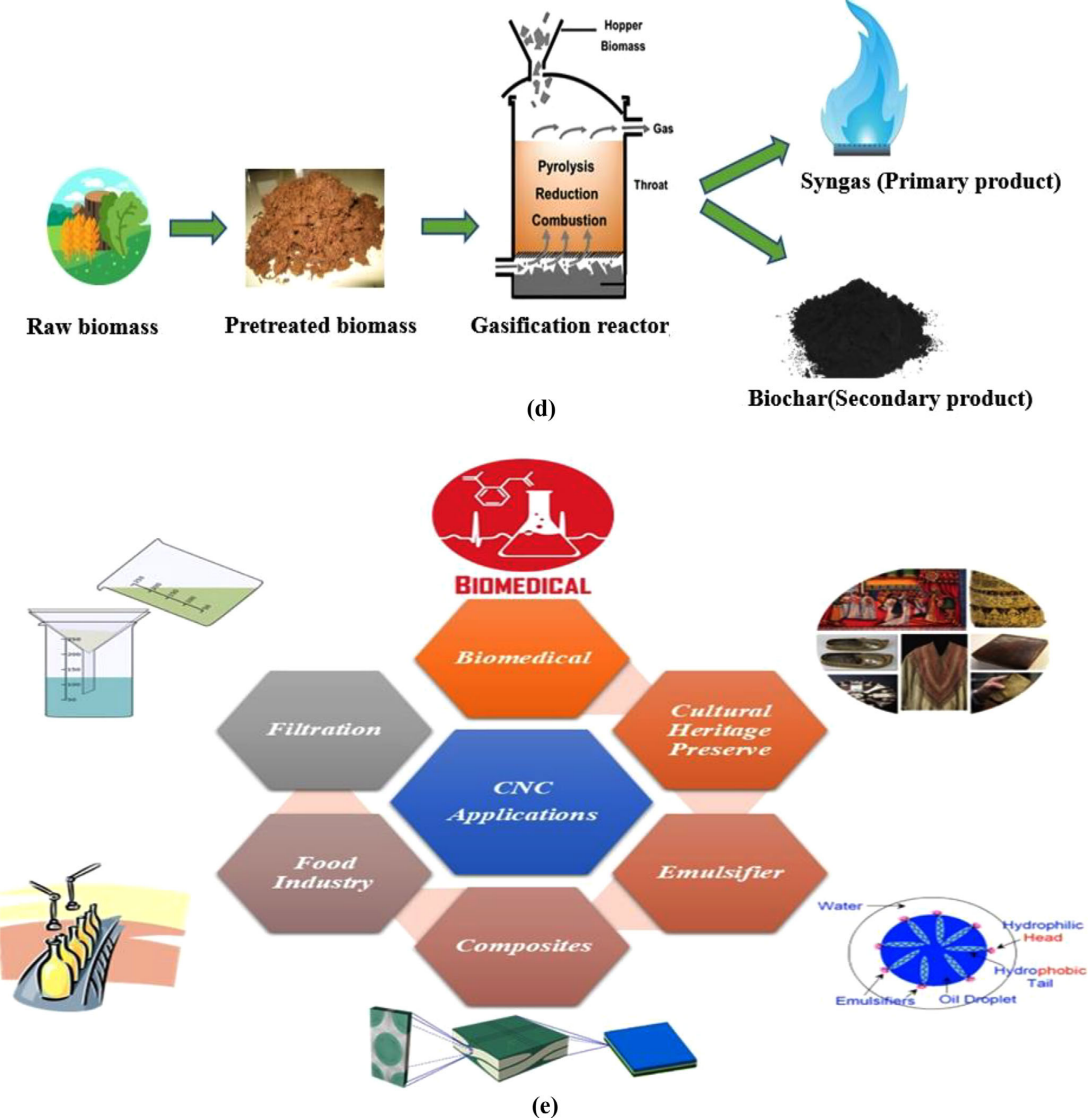

## 3 Production and Activation of Biochar From Agro‐Waste Biomass

### 3.1 Production Process of Raw Biochar From Agro‐Waste Biomass

Biochar, a porous, incredibly carbonaceous solid substance having intensified aromatization, negative surface functional groups, and sophisticated defiance to degradation, comes into existence through the anaerobic decomposition of biomass [88, 89]. Biochar, being eco‐friendly and having tremendous potential uses in different sectors, has been utilized as effluent waste adsorbent, catalytic promoters, soil remediation and composting, energy production, etc. [4, 90, 91, 92, 93]. The conversion of biomass into biochar may undergo several successive steps depending on the type of conversion technologies implemented. In a broad sense, biomass‐to‐biochar conversion technologies technically fall within three categories: (1) Thermochemical, (2) physicochemical, and (3) biochemical, as illustrated in Figure 4a.

Each process mentioned above has its own systematic approach for the production of bio‐char from potential biomass candidates such as agricultural biomass, forestry residue, municipal solid waste, industrial effluents, sewage sludge, etc., containing mainly lignocellulosic and non‐lignocellulosic biomass. Physicochemical conversion of biomass into biochar demands the application of external mechanical stresses for effective and efficient extraction of bioactive constituents [3]. Biochemical conversion requires the active involvement of enzymes and microorganisms encompassing anaerobic digestion, ethanol fermentation, acetone‐butanol‐ethanol fermentation, dark/photo fermentation, etc. Anaerobic bacteria, micro‐organisms capture oxygen from substrate instead of atmospheric air while executing anaerobic digestion, leading to the production of biogas as the main product and bio‐solids as a byproduct. Although considerably slower, the biochemical process demands less external energy as enzymes and microorganisms gradually disintegrate biomass molecules into comparatively miniature fractions [94]. Thermo‐chemical conversion is accomplished through the implementation of pressure, heat, and a catalyst. Thermo‐chemical conversion of biomass into biochar can be carried out employing pyrolysis, gasification, hydrothermal carbonization, torrefaction, etc., as illustrated in Figure 4b **. Pyrolysis**: Pyrolysis refers to an anaerobic non‐oxidative biochar conversion process involving the thermal disintegration of biomass, resulting in the formation of exclusive products such as solid biochar, bio‐oil, and non‐condensable syngas [88]. Based on the variety of temperatures and residence time, the biochar yield varies too. Pyrolysis temperature may vary from 300°C–700°C, whereas the residence time may vary from 1 s to hours with heating rates from less than 11 to 1000°C/s [95]. Based on temperature and residence time of biomass, pyrolysis can be categorized into several types. Such as slow pyrolysis, intermediate pyrolysis, fast pyrolysis, flash pyrolysis, and microwave‐assisted pyrolysis as illustrated in Figure 4c. Slow pyrolysis offers the highest quantity of biochar yield (up to 60 wt.%) with a prolonged residency time of (5 to 30 min or sometimes even 25 to 35 h) at 300°C–550°C [96, 97] producing primary and secondary char. Intermediate pyrolysis occurs within 450°C–550°C taking around 10–30 s for completion, while offering less char yield than slow pyrolysis (up to 40 wt.%) [98]. On the contrary, fast pyrolysis offers 10–20 wt.% char yield at a temperature range of 425°C–1000°C having brief residence times of 0.5–2 s [96]. Flash pyrolysis, on the other hand, offers better yield (28–32 wt%) compared to fast pyrolysis, having a temperature range of 800–1000°C [99]. Microwave‐assisted pyrolysis offers rapid and optimized chemical reactions resulting in almost 30‐wt.% biochar yield. Irrespective of the type of pyrolysis, the products are always solid biochar, liquid bio‐oil, and syngas. While being pyrolyzed, the biomass undergoes successively hemi‐cellulose decomposition at around 200°C–300°C, cellulose decomposition at 400°C, lignin decomposition at 500°C, primary and secondary pyrolysis, and eventually drying for the effective conversion into biochar as illustrated in Figure 4c. **Gasification**: Gasification refers to the thermochemical conversion of carbonaceous biomass into gaseous elements as primary products, such as syngas containing CO_2_, H_2_, CO, CH_4_, and traces of hydrocarbons(biochar) C_2_H_2_, C_2_H_4_, and C_2_H_6_ as byproducts, illustrated in Figure 4d. Before sending biomass to the gasifier, it is made to pass through stages of pretreatment such as steam explosion, milling, acid, alkali, enzymatic treatment, etc. Biomass pretreatment disrupts the lignocellulosic structure recalcitrant, increasing the accessibility of biomass for subsequent processing. Gasification is carried out under reducing conditions in the presence of gasifying agents like oxygen or steam supplied in a sub‐stoichiometric amount at a temperature above 750°C [89, 100]. This thermochemical process is usually carried out following four successive steps: drying, pyrolysis, partial oxidation, and finally reduction of carbonaceous biomass [101]. Fixed bed, liquefied bed, inlet flow, etc., are the commonly used reactors during gasification [100]. Since gasification produces biochar as a byproduct of the thermochemical reaction, the biochar yield is pretty minor. Insignificant amounts of biochar production and subsequent greenhouse gas emissions are the key drawbacks of this biochar synthesis technique [102]. Consequently, this process is not considered ideal for biochar production purposes. This process has been illustrated in Figure 4d. **Torrefaction**: A comparatively moderate thermochemical biochar production technique where biomass is gradually energized thermally at a temperature between 200°C–300°C in anaerobic conditions. Torrefaction is performed with a heating rate being less than 11°C/s while maintaining pressure at atmospheric conditions [103]. Torrefaction enhances biomass material's friability, hydro‐repelling tendency, and susceptibility to bio‐degradation compared to unmodified biomass. This process can alter the properties of biomass, such as moisture content, particle size, surface area, energy density, etc. This process mainly produces solid biochar with a mass yield of 70%–80% (wt.%) and an energy yield of 80%–90% [15]. Torrefied material typically offers an enriched oxygen‐to‐carbon ratio. Also, torrefied material possesses physical and chemical characteristics somewhere in between biomass and biochar, thus making it less suitable to be considered as biochar. Thus, torrefaction is usually employed for the effective removal of moisture content from biomass in order to increase the mass density resulting in the elevation of the heating value of the biomass [104, 105]. **Hydrothermal carbonization**: Biomass material, being significantly moist (having moisture content up to 95%), must be dehumidified before any thermochemical treatment such as pyrolysis [106]. In such a scenario, HTC or Hydrothermal carbonization offers a promising solution in the conversion of wet biomass into biochar. Hydrothermal carbonization refers to a thermochemical biochar production technique that takes place at around 180°C to 250°C in a closed chamber under autogenous pressure [107]. In this process, biomass is blended, mixed with water, and placed inside a reactor where the reactor is gradually energized by utilizing external heating. Once hydrolyzed, products undergo a series of subsequent treatments such as condensation, polymerization, and intramolecular dehydration. Biochar obtained by this process is called hydrochar [108]. High molecular weight and lignin's complicated structural formation have made this process complex. Hydrochar obtained by this process offers an improved hydrogen‐carbon ratio than solid biochar specifications [109]. Finally, the hydrochar is dried up and produces the desired biochar.

### 3.2 Structure, Properties, and Application of Biochar

**FIGURE 5:**
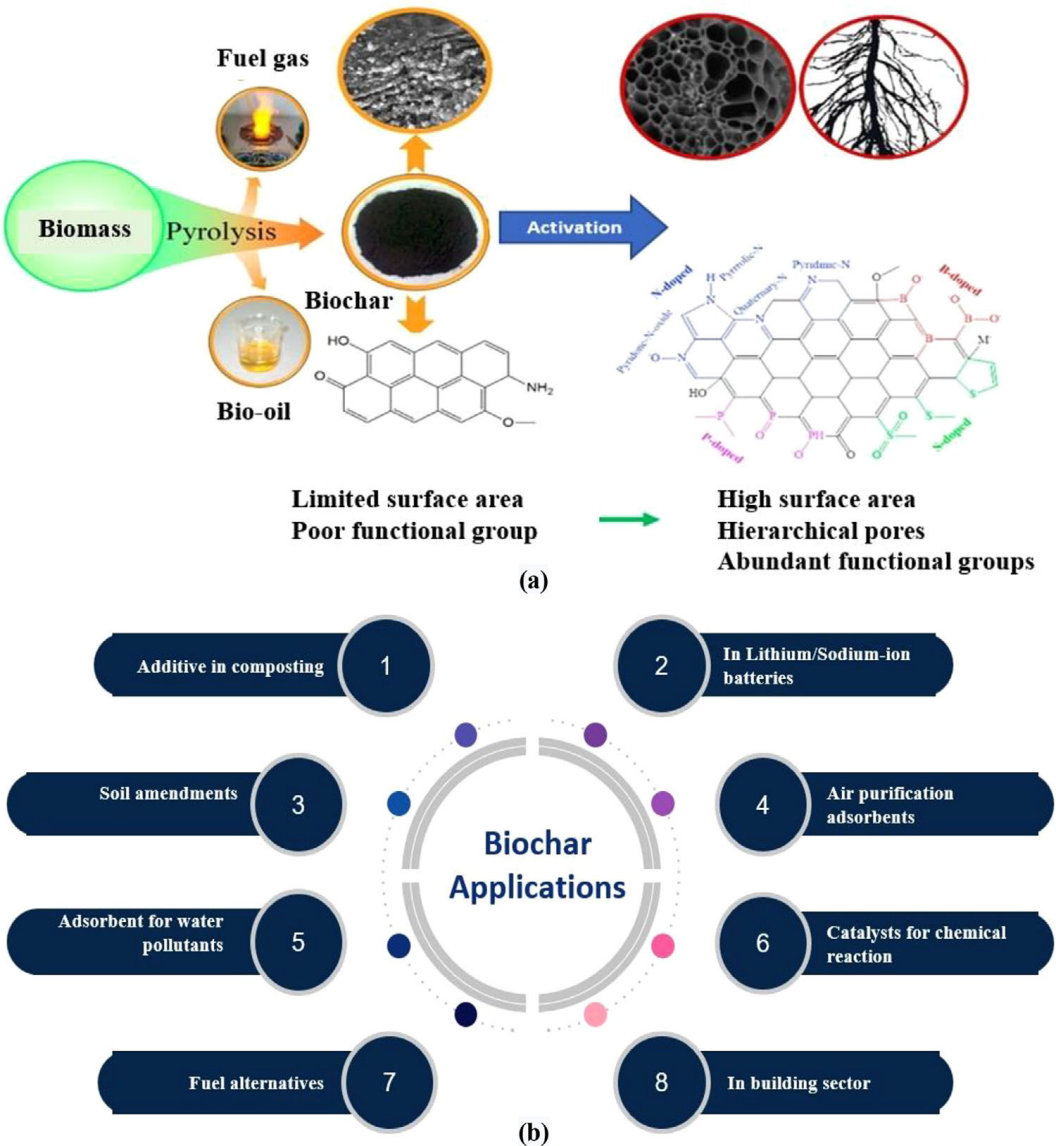
(a) Structural overview of pyrolyzed biochar and its activated form as per the report of [110], and (b) Potential application of biochar in different sectors.

Biochar refers to a pyrolyzed carbon‐enriched material derived from organic biomass through anaerobic thermophysical treatment at elevated temperatures. The existing combination of aliphatic carbon structures and polycyclic aromatic hydrocarbons in the biochar, as well as functional groups such as carboxyl (─COOH), carbonyl (─C═O), hydroxyl (─OH) groups, etc., as illustrated in Figure 5a, plays a critical role in manipulating chemical interactions. Having complex interconnected aromatic carbon networks, biochar usually consists of an augmented surface area, typically fluctuating between 200 and 600 m^2^/g.

Biochar contains countless meso and micropores formed during the pyrolysis of biomass. These pores are the outcomes of scorched residue of organic agricultural waste obtained through pyrolysis. This unique structural feature allows biochar to have numerous uses and applications in various fields [4]. The properties of biochar vary significantly, and this diversity can be identified and illustrated visually when biochar particles are arranged in a loose, granular, loose, or pelleted arrangement. These variations significantly affect parameters such as hydraulic conductivity, porosity, bulk density, specific gravity, water‐holding capacity, specific surface area, particle size distribution, surface charge, etc. [111]. Biochar obtained through anaerobic pyrolysis demonstrates various chemical properties too. Some of these properties are pH measurement, elemental analysis, zeta potential of biochar colloids, etc. [112]. Biochar properties also depend on the pyrolysis method. An example could be fast pyrolysis for biochar synthesis that can develop fine biochar structures with less biochar yield from biomass, having a comparatively lower hydrogen‐to‐carbon ratio. On the contrary, slow pyrolysis develops a larger biochar structure with a higher hydrogen‐to‐carbon ratio [113]. Due to their unique properties of different types, biochar materials often come into use in different applications such as wastewater treatment, air purifications, composting, soil amendments, catalysts for chemical reactions, battery manufacturing, etc., as illustrated in Figure 5b. Having a high affinity toward functional groups of different types, a large surface area, and porosity, biochar can work amazingly as an effective mode of wastewater/effluent treatment alternative. Biochar can effectively capture organic impurities and heavy metal ions from liquids and industrial effluents [8]. The presence of oxygenated groups in biochar (─COOH and ─OH) has illustrated an intensified affinity toward heavy metals [114]. Hence, biochar can be an effective means for wastewater treatment. Biochar can effectively impact in reducing greenhouse gas emissions by adsorbing CO_2_ from the atmosphere. The CO_2_ adsorption capacity of biomass improves with increasing temperature. The use of biochar for the production of biodiesel has massive potential. The production of biodiesel relies significantly on the catalysts, acid density, and surface area. The higher the surface area, the better the biodiesel yield from the production procedure. Since activated biochar offers an increased surface area. Hence, it is a perfect match to be used as a catalyst for biodiesel production. Other than biodiesel production, biochar can play the role of a catalyst in many more chemical reactions. For example, in the Fischer–Tropsch synthesis process, biochar played a significant role as a catalyst for the production of syngas [115] as the product. Biochar works as a soil fertilizing element to enhance its nutritional value. Having numerous pores within the biochar structure, biochar offers water‐holding/storage capacity for a longer period. This significantly reduces irrigation frequency.

### 3.3 Activation Techniques of Raw Biochar

Biochar activation refers to the process of modifying biochar for enhanced characteristics such as porosity, surface area, adsorption capacity, selectivity, applicability, etc. Freshly prepared biochar usually contains many intermolecular pores clogged by trapped gases and volatile elements, leading to tar generation, contracted pore structure, limited surface area distribution, etc. Therefore, biochar activation is necessary to enhance sorption and removal capacity against the selective separation of several toxic pollutants from bulky wastewater bodies [4]. Biochar activation usually falls into two basic types: (1) physical activation, and (2) chemical activation. For better clarity, they are discussed below:

#### 3.3.1 Physical Activation

Refers to a type of biochar activation technique that involves the action of activating agents such as CO_2_, steam, or a combination of both at elevated temperatures to effectively activate adsorbent biochar [116]. Steam is more effective and efficient due to its existing reaction kinetics with carbon compared to other activating agents [117]. The intensity of activation is expressed by the “burn‐off value,” representing the mass disintegration of biochar during the activation process. Higher biochar burn‐off value offers intensified activation. The physical activation method is preferred over the chemical activation method as it ensures minimal material contamination. **(a) CO_2_ activation**: An endothermic biochar activation method using CO_2_ as the activating agent due to its nonreactivity, working between 200°C and 900°C. CO_2_ activation generates new pores within the material as well as expands existing ones [118]. The activation phenomenon initiates with setting up the elevated temperature within the range of 200°C to 900°C. Then, carbon dioxide gas is made to pass over the biochar material. CO_2_ at elevated temperature reacts with carbons within the biochar, producing surface oxide and carbon monoxide, following the Boudouard reaction (CO_2_ + C → 2CO). Formerly, desorption or scavenging of these oxides results in new pore development and enlargement of existing pores within the biochar structure. This is known as dissociative CO_2_ chemical adsorption and desorption. **(b) Steam activation**: An endothermic activation technique using steam for the activating agent to elevate adsorption capacity and to make functional groups readily available so that components with volatility can be ejected with ease [119]. It is engaged in enhancing specific surface area and porosity. Steam activation assists in the eradication of foreign impurities such as organic matter, ash, and incomplete combustion products. Steam activation results in the development of mesopores and micropores where temperature fluctuates between 200°C and 800°C having a heating rate of 5–10°C/min for 30 min to 1 h [120, 121]. Water molecules’ oxygen from steam, upon reacting with the biochar's carbon surface, develops various surface oxides and carbon monoxide. This is referred to as chemisorption. Existing surface oxides get scavenged by carbon monoxide to form carbon dioxide through gasification. The reactions are given in Equations (2)–(4).

(2) C+H2O→CO+H2

(3) 2C+H2→2CH

(4) CO+H2O→CO2+H2

Also, carbon monoxide reacts with water to form CO_2_ and H_2_ gas through a water‐gas shifting reaction. These gases, being driven out of the biochar material, leave behind new pores previously occupied by entrapped gases [122]. Cyclic execution of this process enhances the surface area because of the opening of pores within the carbon structure. Hence, activated biochar with increased surface area and larger pore structures could be produced.

#### 3.3.2 Chemical Activation

In this process, chemical oxidizing agents of different types are introduced to the biochar. It is a single‐step activation technique where the activated biochar is made to pass through intense cleansing for the removal of chemical agents. Sulfonation, oxidation, nanoparticle impregnation, and amination are some of the chemical activation techniques [8]. Some of the chemical activating agents are HNO_3_, H_3_PO_4_, KOH, NaOH, H_2_O_2_, SO_3_H, and ZnCl_2_. The selection of the activating agent relies on the potential biochar application. If the application demands anion adsorption, then it is activated with alkaline agents, offering intensified affinity between adsorbate and adsorbent. Likewise, if the application demands cation adsorption, acid treatment is employed for better adsorption capacity [14]. **(a) Activation with metal composites**: Introducing metal composites can significantly upgrade the biochar material's catalytic activity. Throughout this operation, the solid and gaseous elements of decayed biomass behave as the reducing agents, catalyzing the carbonization of biomass [123]. This improves the properties of biochar and leads to its activation. **(b) Activation with KOH**: KOH is an alkaline activating agent that can significantly upgrade the properties of biomass. Biochar harvested from brewer's spent grain (a byproduct of the brewing industry) when treated with 2 M KOH exhibits increased pore volume, from 0.01 mL/g (untreated) to 8.74 mL/g (treated). Biochar surface area increases from 9.8 to 11.6 m^2^/g KOH activation can offer a carbon yield of up to 73% [124].

**(c) Activation with H_2_O_2_**: Hydrogen peroxide, being an oxidizing agent, can be treated as a biochar activating agent. H_2_O_2_ is capable of working around ambient conditions. Besides, H_2_O_2_ possesses the tendency to degrade back into H_2_O and O_2_. These validate hydrogen peroxide's engagement in biochar activation. H_2_O_2_‐activated biochar shows intensified affinity toward adsorbate material at elevated temperatures. For example, it has been found that at elevated temperatures up to 900°C, grape wood biochar has illustrated an intensified affinity of 99% for herbicides cyhalofop and clomazone [125]. **(d) Activation by potassium carbonate**: Potassium carbonate‐activated biochar has demonstrated excellent biochar characteristics (increased surface area and adsorption capacity) than other non‐activated biochar samples. For the corn stalk, after the completion of potassium carbonate activation, the surface area measured at 750°C was 815 m^2^/g, five times the original surface area of untreated/non‐activated biochar [126]. Similarly, golden shower potassium carbonate‐activated biochar has demonstrated the largest surface area of 1413 m^2^/g at 800°C for 4 h of treatment [127].

## 4 Fabrication of CNC‐FBC‐Based Nanoadsorbents

**FIGURE 6:**
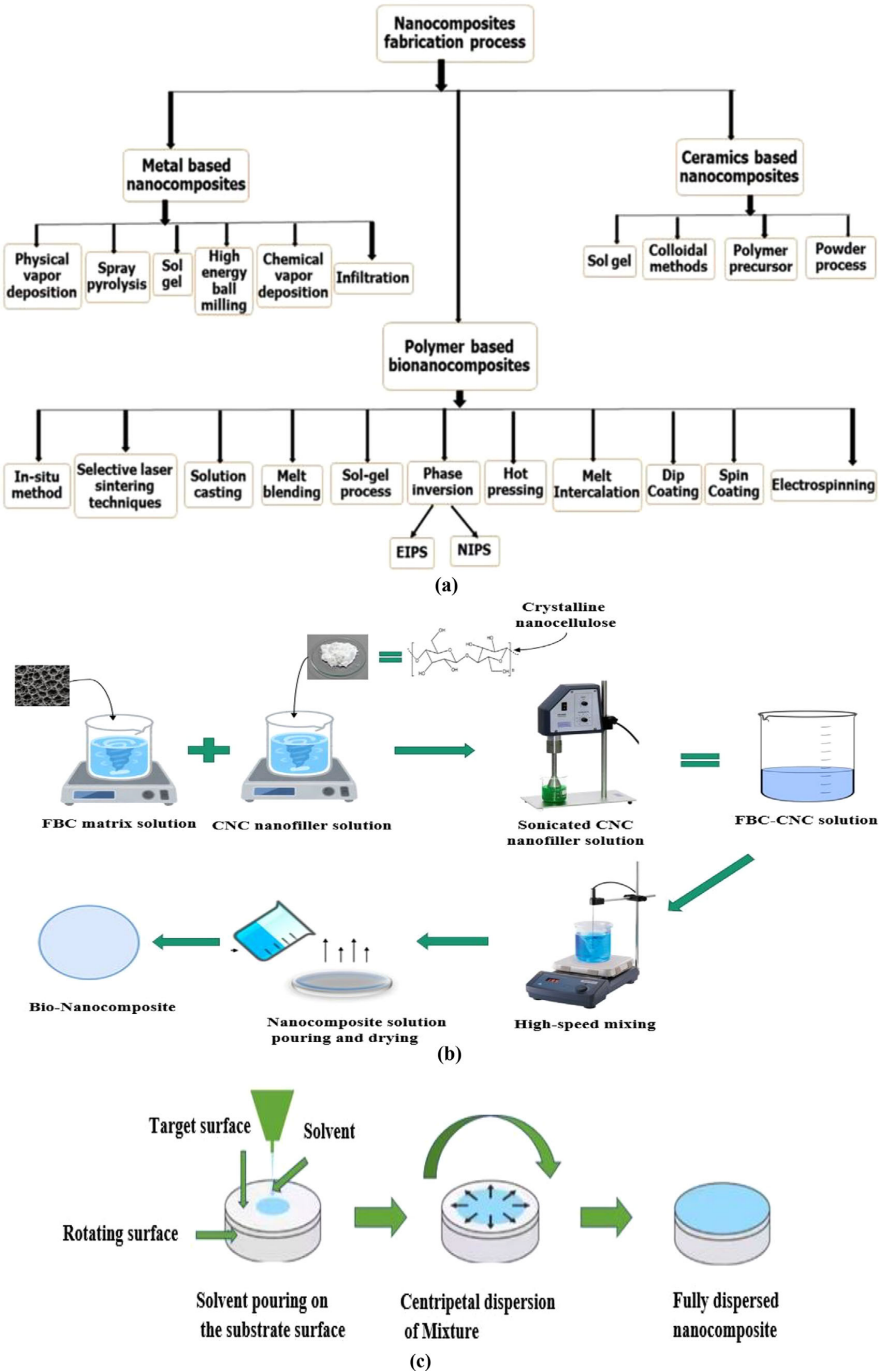
(a) A list of the significant routes/methods of fabrication/production of nanocomposites for various real‐time applications in different sectors, this content is regenerated from [9] with permission from Elsevier, (b) Solution casting method of bio‐nanocomposite fabrication with suitable solvent material, and (c) Schematic of spin coating bio‐nanocomposite fabrication technique.

A nanocomposite is composed of two or more constituents (precursors) that show different physical and chemical properties that cannot be achieved alone by individual constituents. Integrating biopolysaccharide (CNC) with functionalized biochar reveals extended functional and morphological properties, making them highly effective for removing industrial effluent. Biomaterials are being embedded with nanomaterials, developing bio‐nanocomposites. Additionally, bio‐nanocomposites with activated biochar and CNC could possess the advantages of both nanomaterials as well as the availability of different functional groups such as carboxyl (COOH) groups, hydroxyl (OH) groups, amin (NH_2_) groups, etc., making an ideal means of wastewater treatment by effective removal of contaminating heavy metal ions as well as many impurities like dyes, antibiotic residues, pesticide residues, used chemicals, etc, from the considered bulky water bodies [12, 128, 129, 130]. From those, the naturally occurring plant fiber‐derived CNCs and agrowaste biomass‐derived functionalized biochar (FBC) based nano‐adsorbents offer great wastewater treatment potential due to their increased surface areas, mesoporous peripheral structure, honeycomb‐like well‐organized surface microstructure, along with an abundance of active binding sites or functional groups. These CNC‐FBC nanocomposites, often termed nano‐adsorbents, are of significant interest for large‐scale wastewater treatment due to their selectivity, high adsorption capacity, and structural stability. Therefore, by keeping constant the structural integrity, surface morphology, as well as overall properties of the considered agrowaste biomass‐derived CNC‐FBC‐based biopolymeric nanoadsorbents, fabrication/production is very much crucial. Various fabrication strategies have been developed to integrate cellulose nanocrystals (CNCs) with functionalized biochar and polymeric matrices, aiming to ensure uniform dispersion of nanoscale components while preserving structural integrity and accessible surface functionality [131]. Commonly reported methods are illustrated in Figure 6a, and tiny descriptions are included that can be accessed using the associated reference for better clarity and comprehensive analysis.

### 4.1 Solution Casting Method

This cost‐effective approach mixes CNCs and FBC in suitable solvents, followed by drying to produce thin films. It allows control over film thickness, mechanical properties, and surface morphology [12, 132, 133, 134]. This method demands individual activation of both the matrix material (like FBC) and the nanofiller material (such as CNCs). The solution casting method has been illustrated in Figure 6b.

### 4.2 Spin Coating Method

This is a ubiquitous, readily executed method of nanocomposite formation utilizing the physical effect of centripetal force, offering uniform nanocomposite films with controlled thickness. Film properties depend on solvent viscosity, rotational speed, and spin duration. With increasing spinning speed, the material mixture gets dispersed more on the substrate surface, reducing the nano‐composite's thickness. The solvent's viscosity plays a crucial role as a less viscous mixture is more prone to being dispersed throughout. High viscosity is not desired as it inhibits material dispersion [135]. Increased spinning time provokes better dispersion, affecting the nanocomposite material's thickness. For mixtures with a higher viscous consistency, such as gel‐type, spin coating is unsuitable. Hence, this kind of mixture is treated with other techniques for nanocomposite preparation, such as the considered agrowaste biomass‐derived CNC‐FBC‐based biopolymeric nanoadsorbents. This process is illustrated in Figure 6c for better clarity.

### 4.3 Hot Pressing Method

Hot pressing refers to a nanocomposite fabrication technique adopted by the simultaneous implementation of heat and pressure, causing matrix material and external filler to be integrated firmly, as illustrated in Figure 7a. This method does not involve any solvent as both the constituents exist in the solid phase. Having rapid operational time, enhanced interfacial bonds, better densification of the nanocomposite developed, and enhanced thermal and mechanical properties, hot pressing has been preferred over many other available techniques of nanocomposite preparation. By applying heat and pressure to the dry powders of CNCs and FBC, dense nanocomposites are formed with enhanced interfacial bonding, thermal stability, and mechanical strength [136, 137]. That can be beneficially applied in industrial or domestic wastewater treatment for the selective separation of various hazardous toxic pollutants from bulky water bodies by following the fixed‐bed downflow/upflow continuous column adsorption technique for better findings.

FIGURE 7(a) Schematic diagram of the Hot‐pressing process, (b) Electrospinning bio‐nanocomposite fabrication technique regenerated from [138] with proper permission, (c) Sol–gel technique, (d) Phase inversion methods regenerated from the study of [14] with permission from Elsevier, (e) Dip coating method, (f) Melt intercalation process, and (g) In situ polymerization techniques for the fabrication of CNC‐FBC‐based biopolymeric nanocomposites for various uses in several sectors like wastewater treatment.

**Figure:**
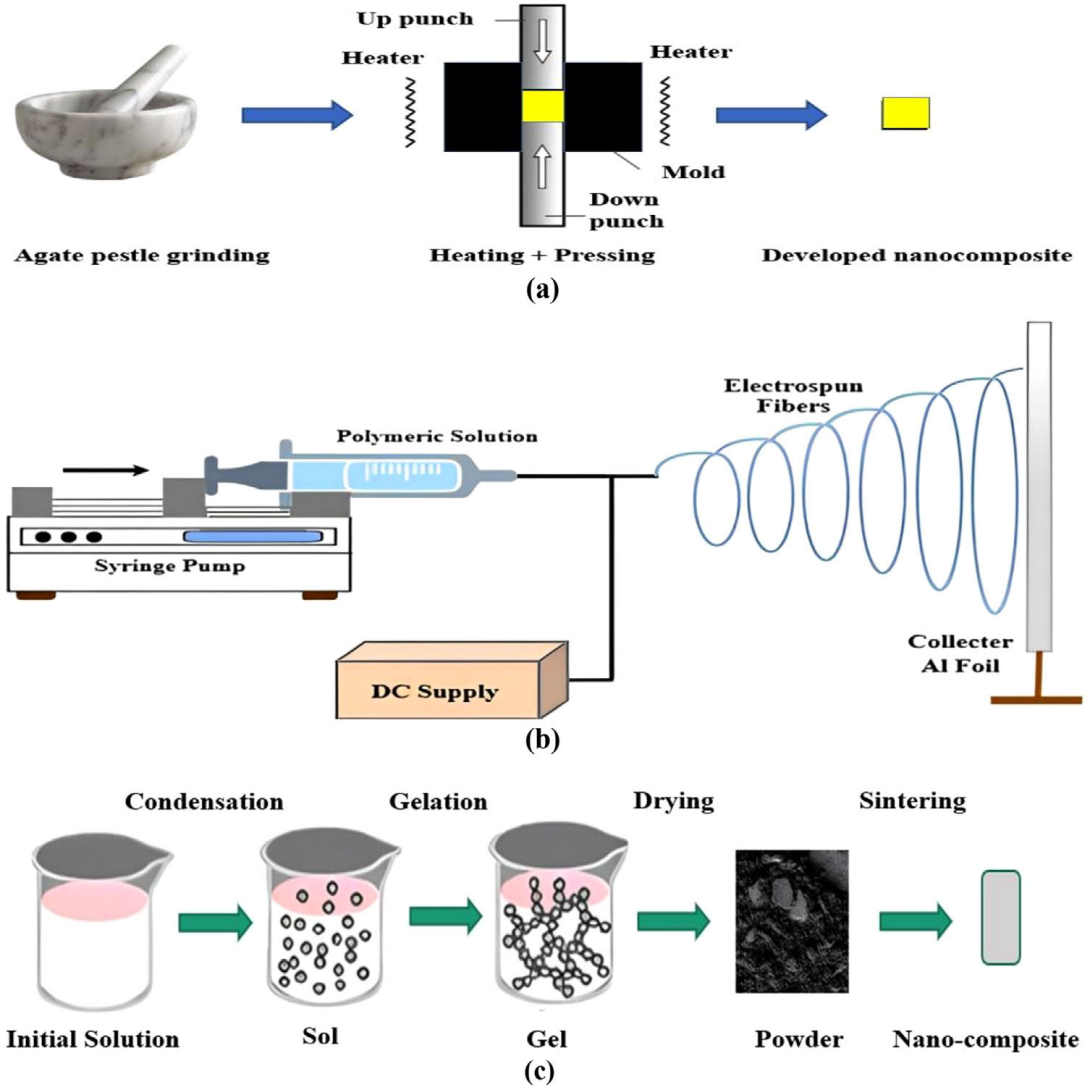

**Figure:**
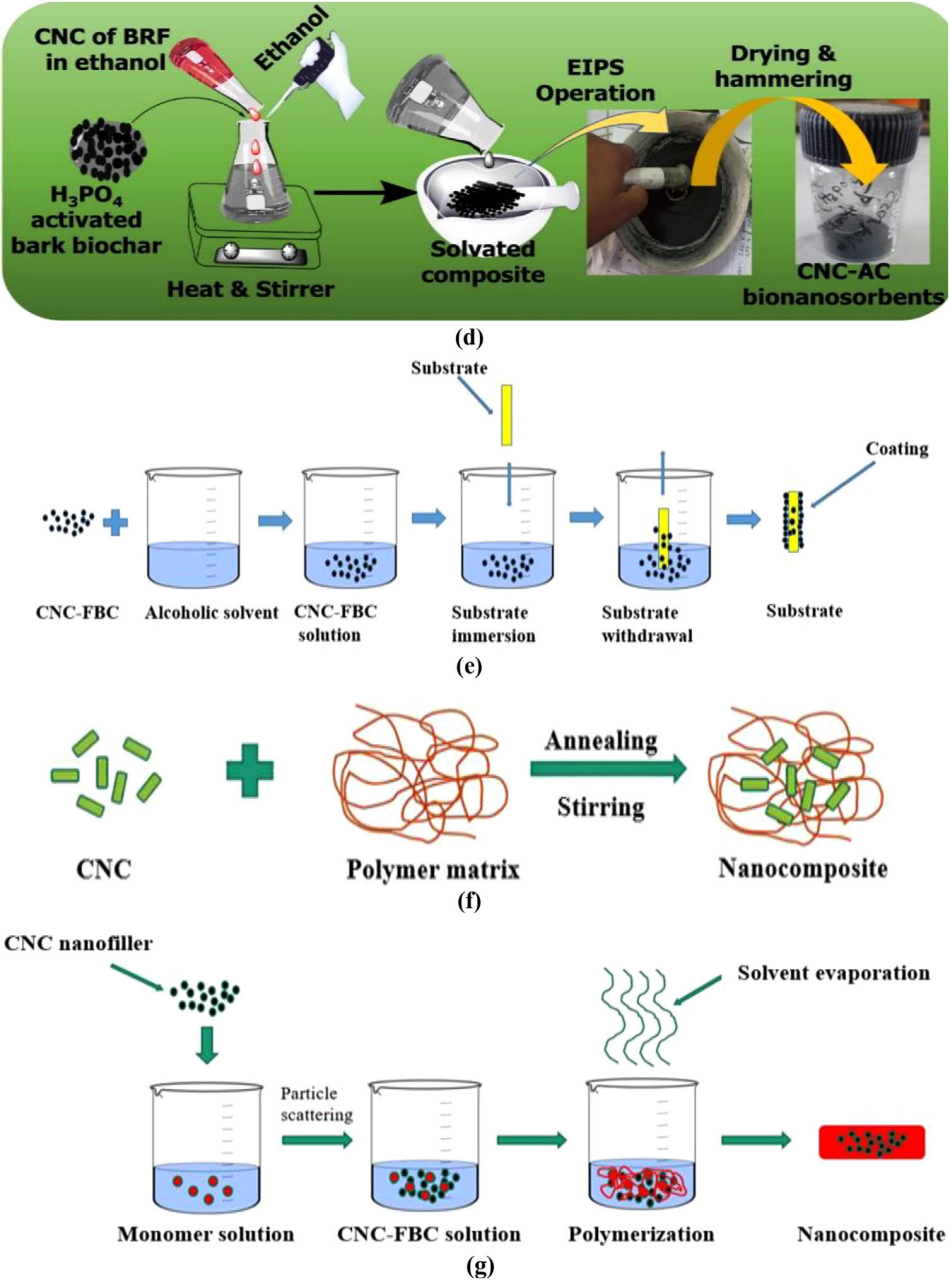

### 4.4 Electrospinning Process

A solution‐based technique that generates ultrafine, porous nanofibers suitable for high‐surface‐area adsorption applications. Precursor dispersion, solvent selection, and voltage control are critical for uniform fiber formation [139, 140, 141]. This technique involves the active participation of an electric charge engaged in crafting fine nanocomposite fibers. This process is casually practiced for the production of nanocomposites having biomaterial‐based fillers. Electrospinning is capable of producing ultrafine nanofiller‐enriched porous nanocomposites, such as the considered agrowaste biomass‐derived CNC‐FBC‐based biopolymeric nanoadsorbents, having tremendous potential usage in many engineering and bulky industrial sectors [139]. A typical solution process is illustrated in Figure 7b. However, for the effective fabrication of the CNC‐FBC biopolymeric nanoadsorbents by conducting this particular electrospinning technique, initially, both the precursors like CNCs and FBC are to be synthesized, collected, modified, and dried to remove the moisture. Then they are incorporated into the liquid phase to make them more homogeneous and uniform; a high‐speed homogenizer and ultrasonic bath could be used. This is followed by the dispersion of both constituents in a suitable solvent material separately. Based on the potential application of the nanocomposite, a convenient polymer solution is prepared as illustrated in Figure 7b. These elements (FBC matrix solution, CNC filler solution, and polymer solution) are later inserted into a hypodermic syringe (spinneret) and then exposed to a high voltage of about 5–50 kV [140]. Loaded material extrudes out through the tip of the syringe as the electrostatic field attracts the material mixture toward a grounded collector. A peristaltic pump is commonly used in electrospinning [141]. During the extrusion of the solution through the syringe tip, the solvent evaporates, leaving behind CNC and FBC‐based nanocomposite fibers. Then, the final yield/nanocomposites could be collected and further processed for direct application in wastewater treatment regarding the adsorptive removal of hazardous toxic pollutants from bulky water bodies by following the fixed‐bed downflow/upflow continuous column adsorption.

### 4.5 Sol–Gel Technique

In the sol–gel technique of nanocomposite fabrication, nanoscale particles are embedded within a solid matrix through a solution‐based wet chemical process, as illustrated in Figure 7c. During this process, a sol (colloidal solid suspension in liquid solvent) gets converted into a gel (semi‐rigid solid 3D network) as nanoparticles are incorporated within the matrix. The sol–gel technique can offer thin nanocomposite film thicknesses of up to a few microns [1, 142]. The process commences with the synthesis and modifications of the preliminary selected precursors. This is followed by the selection of suitable precursors having metal alkoxides. Titanium, silicon, zirconium, and aluminum metal alkoxides are potential inorganic precursors, whereas oligomers and low molecular weight organic compounds are used as organic precursors. Additionally, cellulose, chitin, chitosan, lignin, hemicellulose, CNCs, cyclodextrin, etc, are biopolymeric precursors. Once precursor selection is completed, it is then dissolved in suitable aqueous or alcoholic solvents to make the precursor solutions. The precursor solution is then hydrolyzed, where metal alkoxides are replaced by hydroxyl (─OH) groups. These hydroxyl groups then condense to form small colloidal solid particles in liquid solvents. This is followed by the addition of CNCs nanoparticles within the sol mixture. The sonication, homogenization, or mechanical mixings are carried out for a homogeneously distributed uniform colloidal dispersion of these nano additives in the newly formed complex mixture. The mixture condensation continues, resulting in the development of cross‐linkage among sol particles, which eventually transforms into a viscous, semi‐rigid structure called a gel. The gel formation is then followed by supercritical drying, conventional drying, calcination, or sintering, during which the solvent evaporates, leaving behind the desired nanocomposite materials or adsorbents. For better clarity and to improve their overall physicochemical, thermomechanical, morphological, and microstructural properties, in addition to enlarging their ultimate adsorption capacity, the final yield or CNC‐FBC biopolymeric nanoadsorbents can be further powdered by conducting high‐energy mechanical ball milling and sieved, and then applied via adsorption techniques for the effective treatment of industrial wastewater.

### 4.6 Dip Coating Process

Dip coating refers to a versatile method of fabricating thin film nanocomposites that demands a highly viscous fluid adhesive having a viscosity range of 50–500 millipascals, as illustrated in Figure 7e. This method of nanocomposite fabrication initiates with the preparation of a stable dispersion of CNC‐FBC solution with a suitable solvent, followed by the selection of the substrate materials. The selected substrate must be made to pass through cleaning and necessary pretreatment for effective adhesion during the experimental session. The prepared substrate material is then immersed in the complex mixture of the considered CNC/FBC solution at sufficient speed and residence time, allowing the CNC‐FBC solution to be incorporated appropriately within the substrate material. The substrate is then withdrawn from the solution and set for drying. The post‐nanocomposite production steps could involve the necessary chemical and heat treatment of the newly fabricated biopolymeric nanocomposite materials/bionanoadsorbents, like the considered agrowaste biomass‐derived CNC‐FBC‐based biopolymeric nanoadsorbents, for obtaining the desired material properties [143]. Fabricated nanocomposites quality during this method is usually maintained by the removal speed and the density of the matrix material.

### 4.7 Melt Intercalation Process

This technique doesn't involve any solvent, making it a much‐appreciated sustainable approach toward a variety of bio‐nanocomposite fabrication for several uses in various bulky industrial and engineering sectors [9]. The process commences with annealing or slow external heating of the polymer matrix materials until they melt to a viscous consistency. This is followed by the addition of both preliminary selected precursors, like CNCs and FBC‐based materials, separately and/or simultaneously. However, mechanically intensified stirring is carried out by external shear force through an extruder or internal mixer. It is important to mention that the particular stirring ensures the breakdown of CNCs particle coagulation into the system, which leads to a homogeneous dispersion of the nano‐additives to the matrix melt. Applied shear force to intercalate polymer chains around the considered CNCs and between the FBC layers, which belong to the existing system. The nanofiller distribution is regulated by the peripheral surface of the nanoparticles, the biopolymeric matrix's hosting capacity, and experimental configuration [144]. However, for a better understanding of the scope, contribution, novelty, selectivity, applicability, and significance of this particular melt intercalation method that has usually been conducted for the production of CNC‐FBC‐based multifunctional biopolymeric nanosorbents for various uses in several fields, like wastewater treatment technology, has been illustrated in Figure 7f.

### 4.8 In Situ Polymerization

The in situ polymerization refers to a nanocomposite fabrication technique that involves suitable precursors like CNCs, AC, GO, CS, etc., which are preliminary selected and incorporated with the monomer solution previously prepared having simultaneous application of heating. This process involves intensified blending of the nano‐additives CNCs with the activated FBC matrix materials. Due to the involvement of suitable polymerization initiators or reagent radicals, polymerization takes place gradually around the CNCs nano‐additives. This nanocomposite preparation technique promotes increased interfacial interaction and excellent scattering of the CNC‐FBC, minimizing coagulation to a minimum. Besides, in situ polymerization is considered one of the most effective approaches to controlling particle size and surface microstructure as well as overall morphology of the considered biopolymeric nanoadsorbents [145]. Factors that affect in situ polymerization include polymerization temperature, heating rate, etc, during the processing and production steps. This process is illustrated in Figure 7g for better clarity.

### 4.9 Selective Laser Sintering Method

This method for the fabrication of a bio‐nanocomposite with CNCs and FBC or similar materials includes active dispersion of a powdered polymer sample incorporated with the preliminarily selected and modified precursors like CNCs, AC, GO, CS, FBC, etc, using a convergent laser to execute sintering of the powder particles layer by layer, one after another. Selective laser sintering is considered one of the most advanced support structure/substrate‐free nanocomposite fabrication techniques, offering excellent 3D polymeric nanocomposite molecular arrangement conducted through computer modeling [1]. This process ensures effective fusion of the polymeric FBC matrix material with the CNCs nanofillers by the action of a high‐energy laser for obtaining optimum dispersion. The laser sintering technique is conducted by following three steps. The first step involves raising the temperature of the build and supply chamber to a desired high temperature. This is followed by the fabrication of the part within the build compartment. Finally, the produced nanocomposite is cooled down slowly at room temperature. Once the selective laser sintering is conducted successfully, the produced nanocomposite must undergo the necessary post‐treatment procedures. The selective laser sintering technique is used in various fields of application, such as the aerospace industry, automotive industry, sports industry, healthcare, consumer goods production, pharmacology, and processing industries, due to its superior composite fabrication capability.

### 4.10 Phase Inversion Method

This process refers to a wet chemical nanocomposite fabrication technique that depends on initiating well‐regulated phase isolation within a complex polymeric or biopolymeric solution having the nano‐additives or other filler materials such as cellulose, lignin, clay, chitin, CNCs, AC, MC, GO, rGO, CS, FBC, etc. In a controlled atmosphere, during this process, a biopolymeric matrix changes its phase from liquid to solid through a conversion phenomenon known as demixing, followed by gelation, vitrification, and crystallization [146]. This process initiates with the formation of a solution by dissolving polymer materials like FBC and CNCs within a suitable organic solvent like ethanol. Demixing results in the development of two types of particle stage arrangements within the existing biopolymeric mixture or complex biopolymeric solution. A polymer‐rich and a polymer‐lean stage [9]. This is followed by the addition of a nonsolvent agent that triggers the sedimentation of the mixture of newly generated biopolymeric nanocomposites. Out of these two stages, the stage with high polymer concentration exhibits earlier condensation characteristics compared to the stage having lesser polymer concentration. Unlike the polymer‐rich stage, the polymer‐lean stage produces pores instead of condensation. This leads to the development of a nanocomposite structure having numerous pores within. However, it is important to mention that, based on the main parameter/influencing factors of the experimental operations of the phase inversion method, this particular technique has been classified into four different groups. Such as: (a) Non‐solvent‐induced phase separation (NIPS) technique; (b) Thermal induced phase separation (TIPS) technique; (c) Vapor induced phase separation (VIPS) technique; and (d) Solvent evaporation induced phase separation (EIPS) technique. However, for better clarity, a flow diagram has been shown in Figure 7d, which was conducted for the fabrication of CNC‐AC bionanoadsorbents for wastewater treatment technology via fixed‐bed continuous column adsorption mode of operation for the selective separation of the emerging toxic pollutants from bulky water bodies as per the report of Rahman and co‐workers during their research work.

In conclusion, each fabrication route presents effectiveness in terms of structural control, energy requirements, and industrial feasibility. Solution‐based methods, including solution casting and sol–gel processing, rely on solvent‐assisted mixing to achieve homogeneous distribution of CNCs within the matrix. Although these techniques may introduce limitations related to solvent handling and scalability, they can be effective for precise control over microstructure. In contrast, solvent‐free approaches such as melt processing and thermal compression utilize heat and mechanical forces to promote interfacial interactions between components. These methods are considered more suitable for large‐scale production due to reduced solvent use and improved process efficiency. The selection of a suitable method depends on the desired balance between performance and scalability. The aforementioned methods can be applied depending on the targeted application, required nanocomposite properties, and scalability considerations.

## 5 Potential Characterization Techniques

The structural and functional properties of CNC–biochar nanocomposites are typically evaluated using a combination of spectroscopic, microscopic, and thermal analysis techniques. These methods provide insights into surface chemistry, morphology, crystallinity, and thermal stability. Rather than focusing on procedural details, it is important to emphasize that characterization techniques play a critical role in linking material structure to adsorption performance and potential. Comprehensive descriptions of experimental protocols are available in previously published reviews and are therefore not repeated here. Hence, this section has been dedicated to describing the scope, contribution, significance, key outcomes, selectivity, and applicability by providing required scientific discussion, logical argument, figures/tables/illustrations, and examples for better clarity.

### 5.1 X‐Ray Diffraction Analysis

This is a very rapid nondestructive analytical technique that helps to study chemical structure, composition, crystalline nature, particle size, lattice space, etc., of the subjected biopolymeric samples or other related samples [147, 148, 149]. XRD lets the sample be scanned in a 2θ range varying from 5° to 80°. The spectrum is established from the measurement of subjected samples of raw fiber, alkali‐treated fiber, bleached fiber, CNCs, and CNC‐FBC biopolymeric nanocomposites, which explains the diffraction pattern of crystalline and amorphous areas separately as per earlier literature [5].

### 5.2 FTIR Analysis

FTIR (Fourier Transform Infrared Spectroscopy) analysis, a nondestructive analytical method, of the subjected CNCs functionalized bio‐nano adsorbent provides information about absorption peaks, their positions, and chemical vibrations of present functional groups in the sample. So, the presence or absence of a functional group, the purity of the subjected sample can be analyzed by this method [5, 150]. Notably, in Figure 8B and Table S1 represent a list of the most widely present functional groups with their particular absorption peaks, type of bond vibration, intensity, and locations that have generally been observed in FTIR spectra of CNC and natural lignocellulosic fiber‐based multifunctional biopolymeric nanoadsorbents. In some cases, the absorption peaks might show traces of functional groups that are not directly related to the lignocellulosic fiber that's found in plants (like S–H, S–S, NO_2_, etc.). This might be due to the chemical modifications of fibers during the experimental session [5].

FIGURE 8Comparative analysis of various biopolymeric samples such as lignocellulosic fibers at different stages and their composites. (A) XRD analysis reproduced from the study of [46] with permission from Elsevier, (B) FTIR‐ATR analysis of RF, ATF, BF, ANC, CNC‐AC bionanoadsorbents before as well as after adsorption as per the study of [14] with permission from Elsevier, (C) Solid state ^13^C NMR spectra of α‐cellulose (a), amorphous cellulose (b), “crystalline fraction of cellulose”(c) as per the study of [151] with permission, (D) Thermal analysis of (a) raw fiber, (b) alkali‐treated fiber, (c) bleached fiber, and (d) CNCs of sugarcane leaf sheath fiber (SLSF) as per the study of [46] with permission from Elsevier. (E) Comparative morphological analysis of several biopolymeric samples, such as (a) RF, (b) ATF, (c) BF, (d) CNC, (e) CNC‐MC nanoadsorbents (before adsorption), as well as (f) CNC‐MC nanoadsorbents (after adsorption), as per the FESEM observation of [12], this content is reproduced with permission from Elsevier. (F) XPS spectra of the PEI‐silica nanoparticles (a), curve‐fitting for C 1 s (b), and N 1 s (c), according to the study of [152] with permission, and (G) BET analysis of a FRHBC nanosorbents including N_2_ adsorption–desorption isotherm curve as per [4]. This content has been reproduced with permission from Elsevier.

**Figure:**
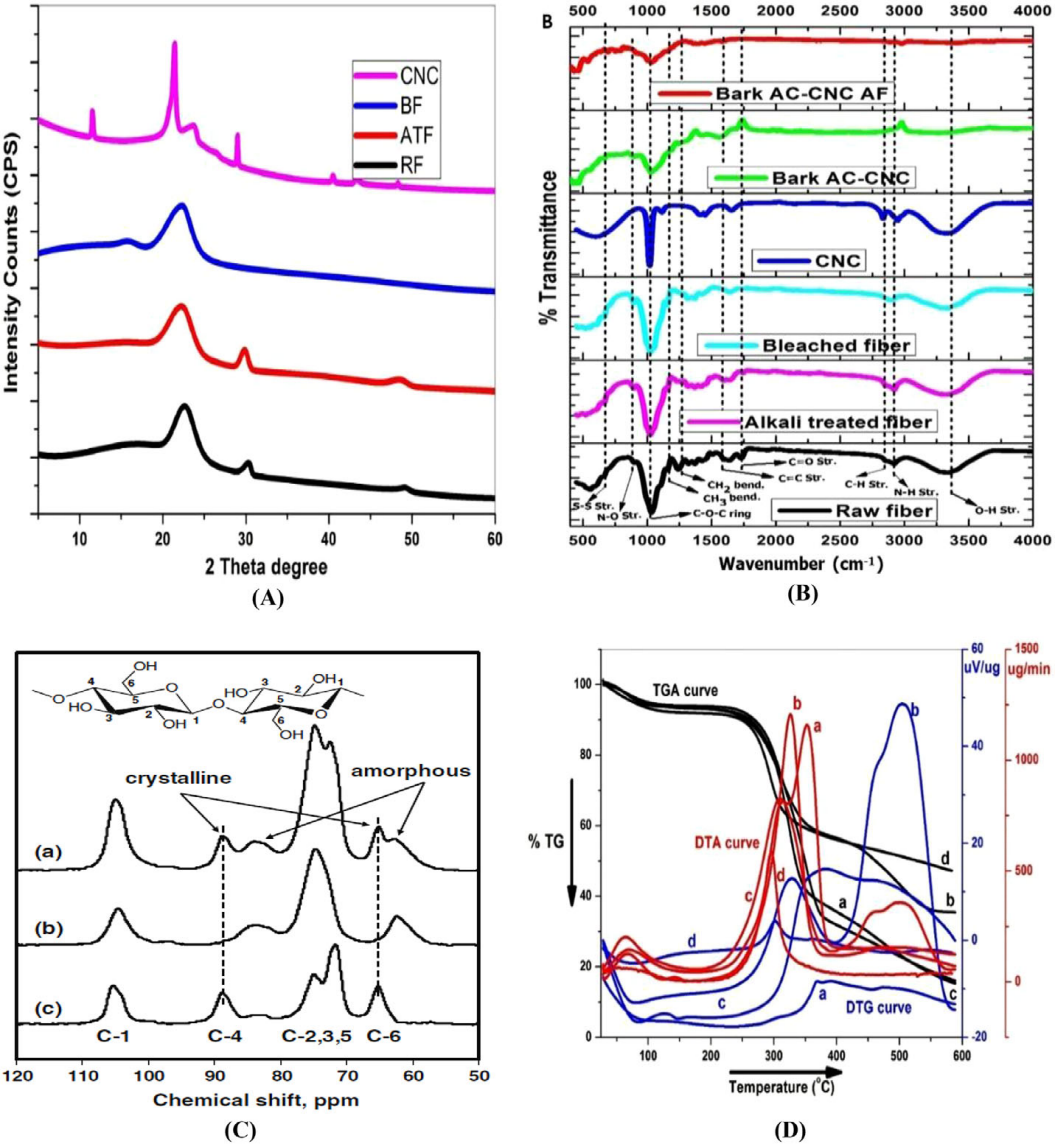

**Figure:**
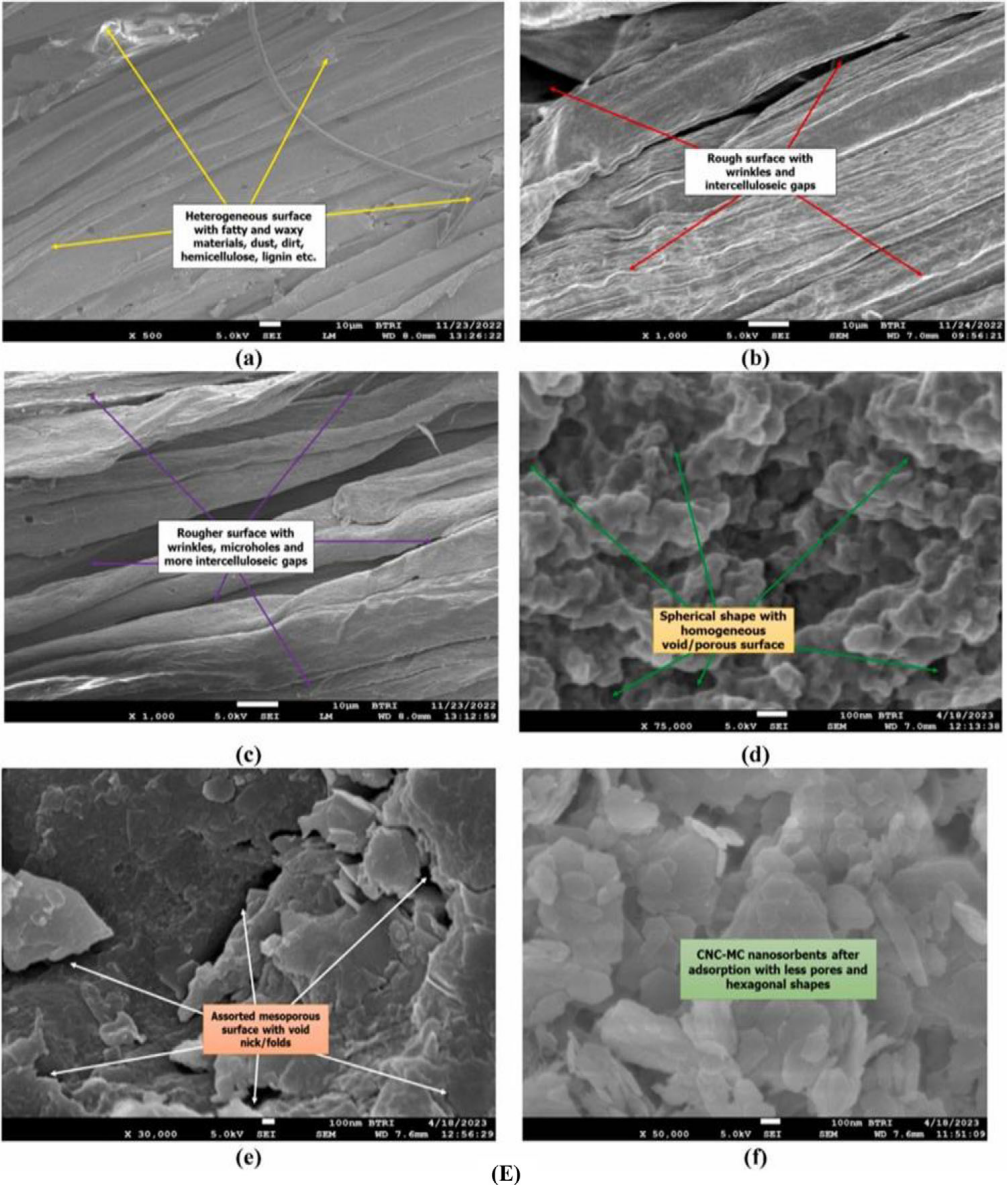

**Figure:**
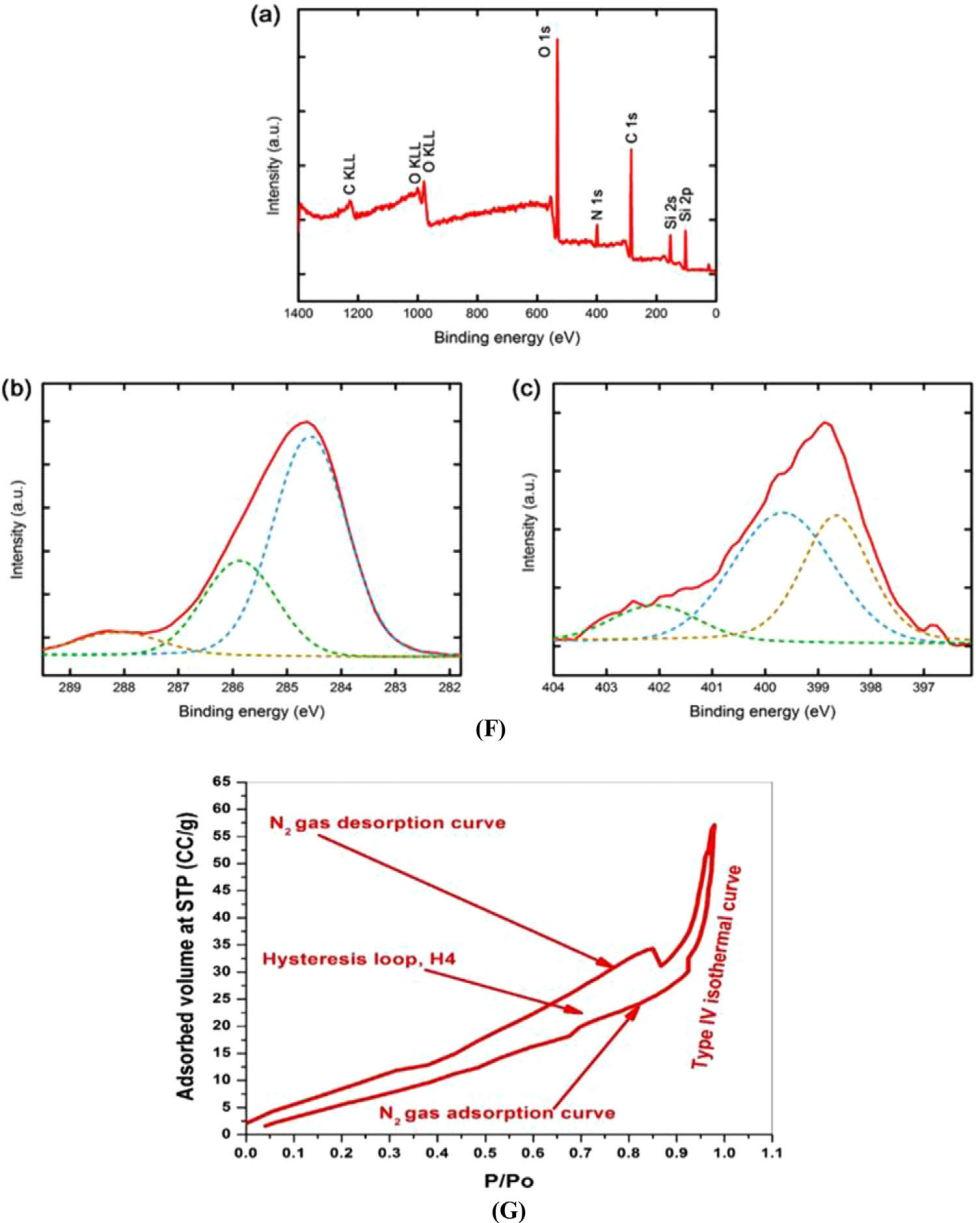

### 5.3 ^13^C Cross‐Polarization Magic Angle Spinning Nuclear Magnetic Resonance Analysis

This is a highly valuable nondestructive method that provides detailed information on molecular structure and differentiates between crystalline and amorphous regions by applying Gaussian and/or Lorentzian fitting for peak convolution [153]. For understanding structure and property relationships, molecular interactions, crystallinity, and actual chemical composition in CNC‐loaded functionalized biochar nanoadsorbents and related samples, ^13^C CPMAS NMR is an eloquent tool. This specific sophisticated technique could provide valuable insights into carbon atoms, their bonding and chemical environment, molecular structure, and dynamics of crystalline and especially amorphous regions that belong to the considered biopolymeric samples, focusing on the peak shape, intensity, and chemical shifting [154, 155, 156, 157]. However, it is important to mention that the naturally occurring celluloses possess two native varieties, like I_α_ and I_β_ cellulose, whereas the crucial influences that describe a precise inherent cellulose are the size of the microfibrils, as well as the percentage of each form individually. These proportions could be recognized by discrete structures that have been observed in solid‐state ^13^C NMR spectra, particularly the peaks connected to the C_4_ resonance, as well as to a reduced extent, those at C_6_ and C_1,_ respectively. By classifying the particular carbon shifts linked to the functional group (such as C–O–C in the considered cellulose), this specific characterization method can confirm the chemical variations or structural variations that are included through the physicochemical modification/complexation during experimental sessions. For a better understanding, an example of solid‐state ^13^C NMR spectra of α‐cellulose (a), amorphous cellulose (b), “crystalline fraction of cellulose” (c) has been shown in Figure 8C. Noteworthy that, the NMR techniques could be beneficial to detect the exchanges between cellulose/CNCs and the incorporated nano filler/additive materials in the structure of the newly produced biopolymeric nanoadsorbents additionally it also helps to understand the interaction between the applied biopolymeric nanosorbents and metal ions or other functional groups, that could be much more useful in understanding the surface chemistry modifications for enhanced adsorption activity against the selective separation of several hazardous pollutants from bulky wastewater bodies [3].

### 5.4 Thermal Analysis

Thermal analytical techniques like TGA/DTA/DTG are commonly used to quantify the change in the subjected polymeric/biopolymeric/other related material mass/weight as it is heated in a controlled environment during the characterization or experimental session. This destructive form of testing provides information on chemical composition, thermal decomposition properties (melting temperature, polymer relaxation temperature, onset temperature, maximum decomposition temperature, quantity of deformation at a specific temperature range, phase change, etc.), products released with varying decomposition temperature, materials stability at different temperatures, energy need for occurring decomposition, residual mass etc. as a function of increasing temperature up to 1000°C [9, 158].

### 5.5 Field Emission Scanning Electron Microscope (FESEM) Analysis

This (FESEM) characterization technique helps to estimate the peripheral surface morphology and topography, indicating surface microstructure, cracking, and wrinkle of CNC‐loaded functionalized biochar‐based nano adsorbent samples. For performing the analysis of the composite samples, they could be attached to aluminum stubs by carbon tape, then sputtered with gold/platinum/carbon coating to make it more conductive for creating a good quality, high‐resolution image [159]. However, it is also important to mention that the FESEM analysis can also detect the micro and nanostructure, porosity, particle size, surface roughness, structure type, shrinkage, sorption profiling, etc. The nanoparticles histogram and their size distribution curve of the considered bionanoadsorbents can also be measured from the obtained FESEM micrograph [5, 160]. For better clarity, go through Figure 8E, as per the FESEM observation of [12]. They have conducted their research work for the selective separation of both toxic heavy metals and hazardous dyes from the industrial wastewater by applying CNC‐MC‐based multifunctional biopolymeric nanoadsorbents via a fixed‐bed down‐flow continuous adsorption study for better findings.

### 5.6 XPS Analysis

X‐ray photoelectron spectroscopy (XPS), also known as ESCA (Electron Spectroscopy for Chemical Analysis), is a surface‐sensitive analysis that provides important information on nanostructured natural or synthetic materials. It is a well‐established method for characterization (in this case, bionanoadsorbent) as it provides chemical, elemental, and quantification analyses on the sample's surface [1]. It provides information regarding surface layer, coating thickness and structure, molecular orientation, surface functionality, particle size up to the nanometer scale that can't otherwise be readily measured by other analysis techniques. As in the case of nanostructured materials, surface properties (actual composition and chemical state of surfaces) are the key controlling characteristics of material properties. XPS here can provide information about chemical state, actual composition of surface and interface that dominate nanomaterial properties [161]. However, for better clarity, an example of XPS analysis (addressing nanoadsorbents) has been shown in Figure 8F.

### 5.7 BET Analysis

Brunauer–Emmett–Teller (BET) analysis measures the pore size, total pore volume, and specific surface area of the subjected bio‐nano adsorbent using a BET sorptometer. Via nitrogen gas adsorption, the surface area of a sample of varying porosity is determined as a function of relative pressure. Findings of the BET analysis of FRHBC (functionalized rice husk‐derived biochar) are represented in Figure 8G. The analysis result indicates a mesoporous structure with high specific surface area and average pore diameter compared to previously reported literature [162, 163]. It can be concluded that higher specific surface area, average pore diameter, and pore volume of the subjected adsorbents are more conducive to enhanced sorption capacity for heavy metal and dye molecules from industrial effluent.

### 5.8 Wastewater Analysis

The presence of most of the metallic and heavy metal ions (Cu, Ni, Co, Cd, Zn, Mn, Pb, Hg, Cr, As, Fe, Al, Ca, Mg, etc) can be detected by AAS (atomic absorption spectroscopy). This method involves the absorption of photo radiation by heavy metals in the gaseous state at the ppb to ppm level [8, 164]. It is important to mention that the AAS technique comes with some limitations, such as it can detect only one element at a time, has low sensitivity, failure to detect molybdenum and silicon due to their tendency to form oxides, etc. [165]. For the detection of organic and inorganic dyes, UV‐NIR spectroscopy (ultraviolet near infrared spectroscopy) is used, which works on the principle of the Beer–Lambert law [166]. Some contaminants like pesticide residue, antibiotic residue, and microplastic residue are detected by following analysis methods like gas chromatography‐mass spectrometry, liquid chromatography, Raman spectra, optical microscopy, and Fourier transform infrared spectra, respectively [166, 167, 168, 169, 170, 171].

### 5.9 Experimental Data Analysis

Notably, the actual performance of the fixed‐bed continuous column adsorption would be progressively measured by applying the significant breakthrough curve (BTC) concept regarding the real‐time adsorption experimental data of a particular system. The BTC curve could be attained by plotting the ratio of the equilibrium concentration (denoted by C_f_) and the initial concentration of the feed solution (denoted by C_i_) (*i.e*., C_f_/C_i_,) against time (t, minutes) (shown in Figure 9A).

FIGURE 9(A) Nonlinear fitting on the experimental BTC curve of the selective separation of Ni^2+^ and Eosin Y dye from the wastewater bodies onto the peripheral surface of the chitosan‐coated bentonite clay biopolymeric nanoadsorbents via fixed‐bed column adsorption according to the study of [172] with permission from Elsevier. (B) Proposed synergistic mechanism for the treatment of bulky industrial wastewater using CNC‐loaded functionalized biochar‐based nanoadsorbents, illustrating processes such as deep penetration, intra‐particle diffusion, physisorption, and chemisorption.

**Figure:**
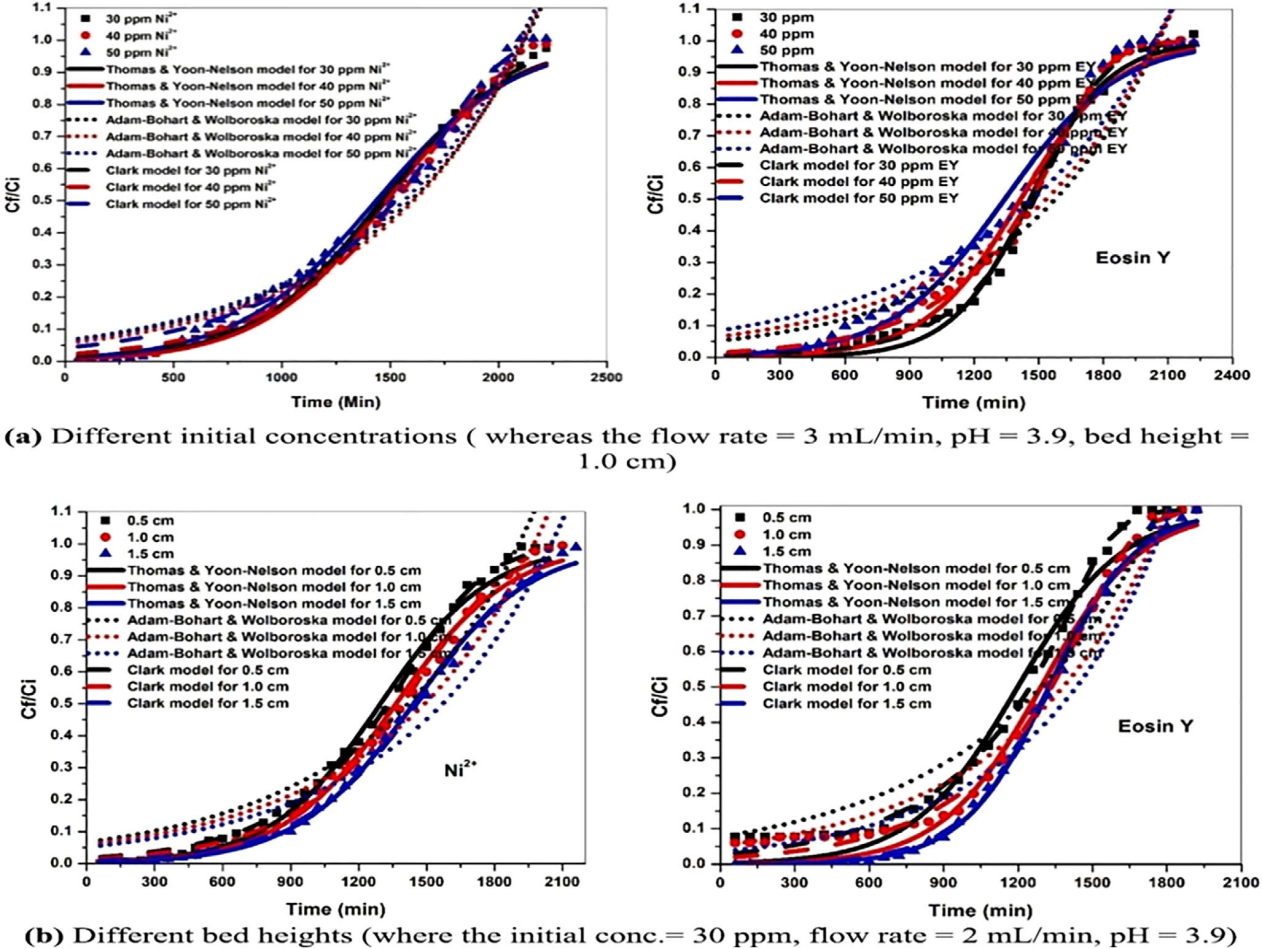

**Figure:**
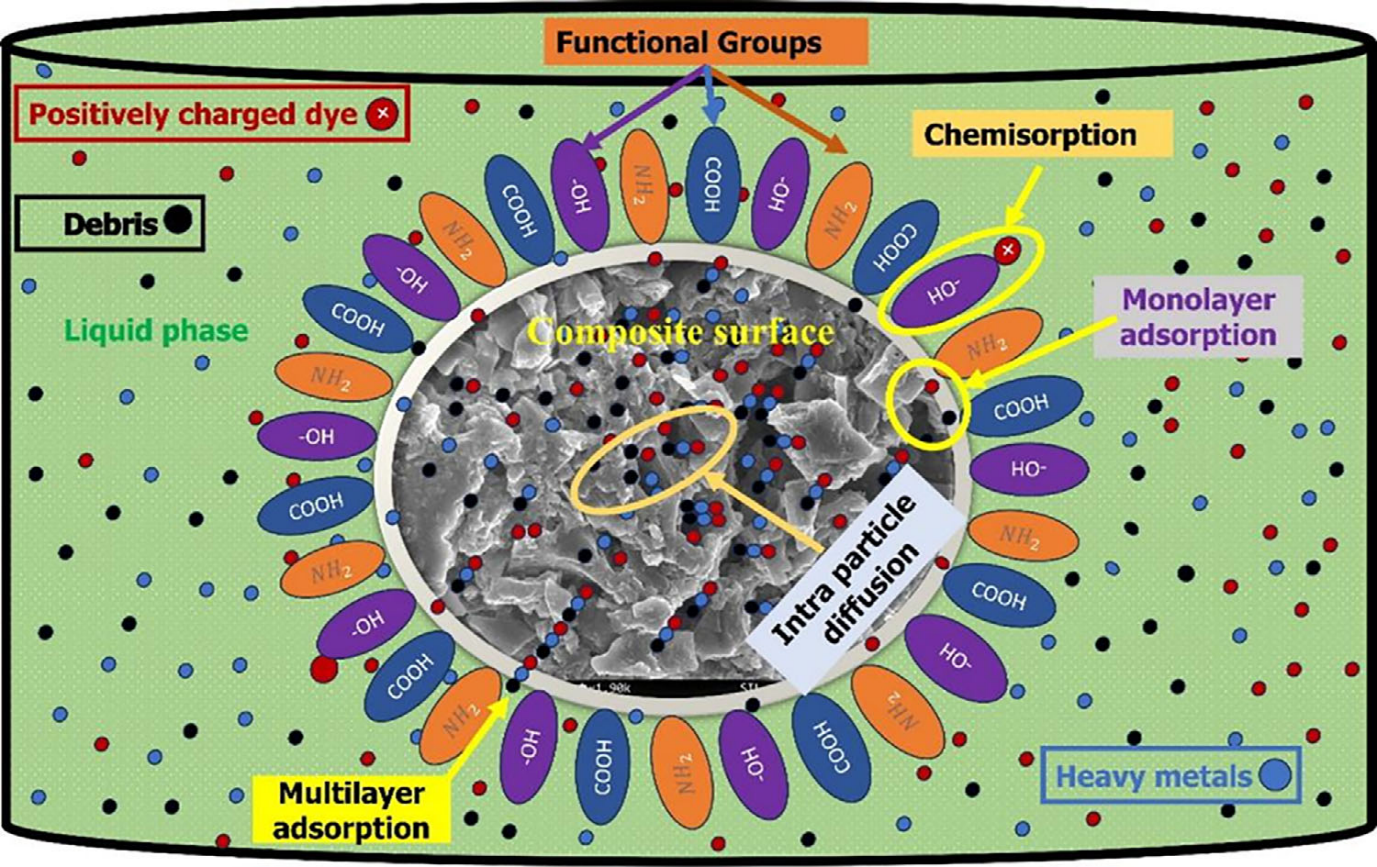

## 6 Application of CNC‐FBC‐Based Nanosorbents on Industrial Wastewater Treatment

There are numerous separation techniques that are commonly applied in the industry for purifying effluent. Although these techniques, coagulation–flocculation, various filtration methods—including sand, multimedia, and membrane processes, chemical treatments, ion exchange, biological processes, and electrocoagulation, are less influential as they either generate hazardous byproducts or consume substantial energy, resulting in high operational cost and lowering environmental standards [173, 174]. Among these, adsorption is recognized as a highly efficient and versatile method, capable of removing a wide range of pollutants with minimal sludge generation and lower energy requirements [175, 176]. It avoids excessive chemical use and toxic byproduct formation compared to conventional methods and does not require frequent regeneration like ion exchange [177]. Adsorption occurs via physical interactions (e.g., van der Waals forces, hydrogen bonding) or chemical bonding (e.g., ionic or covalent), influencing reversibility and removal efficiency [1, 2, 178]. Its performance depends on adsorbent properties such as surface area, pore structure, and operating conditions. While batch adsorption is suitable for small‐scale applications, it faces limitations in scalability and operational efficiency [9, 179]. In contrast, continuous fixed‐bed column systems enable steady operation, higher throughput, and consistent performance, making them more suitable for industrial applications [4, 180].

### 6.1 Fixed‐Bed Column Adsorption Study

The materials, CNC‐FBC nanoadsorbents, are particularly effective in fixed‐bed continuous column systems, where their mechanical stability and flow properties enhance large‐scale wastewater treatment efficiency [3, 12, 132]. CNC‐FBC nanoadsorbents are highly versatile for the selective removal of pollutants from industrial and municipal wastewater, including heavy metals, dyes, and organic contaminants. Their effectiveness is largely attributed to the presence of abundant functional groups and porous structures that facilitate adsorption. These key features, high surface area, porosity, and abundance of functional groups, facilitate the adsorption of heavy metals (e.g., Cu, Pb, Cd, Cr), dyes, antibiotics, pesticides, and other emerging contaminants. Beyond adsorption, CNC‐FBC composites are explored in catalysis, membrane technology, and sensor applications, benefiting from their tunable morphology and chemical functionality. Their biodegradability and eco‐friendly nature make them suitable for sustainable wastewater management strategies. Scalability is supported by multiple fabrication methods, allowing adaptation to both laboratory and industrial‐scale operations. Overall, CNC‐FBC nanoadsorbents represent a practical, high‐performance, and environmentally compatible solution for complex wastewater remediation [5, 9, 146]. However, reported efficiencies are often obtained under controlled laboratory conditions using single‐component systems. As a result, these findings should be interpreted with caution when considering practical applications. For better clarity, a sketch diagram addressing the experimental setup of a fixed‐bed downflow column continuous adsorption study in a lab scale/piloting for wastewater treatment has been shown in Figure S1.

### 6.2 Desorption Study for Reusing the Nanoadsorbents

Desorption studies are of utmost importance when considering this aspect of the use of wastewater, as they encompass the sustainability, cost, and feasibility of using adsorption technology in an industrial context. Although adsorption can effectively remove pollutants, practically, its ongoing use depends on the regeneration of the adsorbent, where the adsorbent is reused for future adsorptive events without significant loss of efficacy. This is most important in large operating systems, such as fixed‐bed columns, where frequent replacement of adsorbents is generally uneconomical and not environmentally viable. If a proper desorption is achieved, there should be sufficient efficiencies in the active sites of the adsorptive surface to allow reuse, which should minimize overall waste of the material, reduce operational costs, and lessen the environmental impact of the treatment system. A recent study shows [181, 182, 183, 184, 185] a nanocomposite adsorbent ZnO‐TiO_2_/rGO (zinc oxide‐doped titanium dioxide/reduced graphene oxide) that was created via a two‐step hydrothermal method to treat water contaminated with the organic pollutant, namely, methylene blue (MB) dye. The cationic dye (C_16_H_18_ClN_3_S) was selected as a model pollutant of textile wastewater because of its toxicity and persistence. The nanocomposite achieved 43.68% adsorption of MB in 60 min (pH 9, dark conditions) and 99.84% total removal after 60 min of UV irradiation, which was attributed to the synergistic effect of adsorption‐photocatalysis enabled by electrostatic attraction, π‐π interactions, and radical‐driven degradation. On the other hand, fly ash‐TiO_2_ nanocomposite has simultaneously removed material from wastewater (by adsorption and UV‐stimulated photocatalysis) at >90% copper (Cu^2^^+^) and industrial dyes (Bemacid Blau/Rot) while utilizing waste fly ash to develop a cheaper multi‐pollutant treatment [186]. Graphene‐TiO_2_ and graphene‐ZnO nanocomposites are remarkably efficient at both adsorption and photocatalytic degradation of priority wastewater pollutants. Both systems can achieve >90% removal of organic dyes (methylene blue, methyl orange, crystal violet, rhodamine B) and phenols (e.g., complete removal of 2‐chlorophenol at pH 12; 96.4%, respectively), and total inactivation of >99% pathogenic bacteria (e.g., E. coli) under solar/UV irradiation [187]. Meanwhile, CNC‐loaded functionalized biochar nanoadsorbents provide significant advantages for industrial wastewater treatment. Despite the promising laboratory‐scale performance of CNC‐loaded functionalized biochar nanoadsorbents in fixed‐bed column systems, several challenges hinder their direct translation to large‐scale industrial applications (shown in Table S2).

### 6.3 Comparative Study

Toxic substances like dyes, phenols, heavy metals (lead, chromium, cadmium, and nickel) are common pollutants that may be present in industrial effluent/wastewater. CNC‐loaded functionalized biochar nano‐adsorbents are a highly efficient, sustainable, and cost‐effective solution for removing pollutants. As biochar is functionalized and combined with CNC, it shows exceptional efficiency in removing pollutants from bulky wastewater bodies. It has a tremendous adsorption capacity. Biochar, produced by the pyrolysis of biomass, is known for its high surface area, porosity, and affordability. When functionalized and loaded with CNC, it gains additional active sites and enhanced mechanical properties, making it a potent adsorbent for the selective separation of various pollutants from industrial effluents [3]. For instance, the hydroxyl (─OH) and carboxyl (COOH) groups of CNC can form complexes with heavy metals via a chemisorption mechanism, while the porous structure of biochar facilitates mono and multilayer physisorption. In this study, the effectiveness of CNC‐loaded biochar‐based nanoadsorbents in industrial effluent will be understood. The integration of CNC into functionalized biochar offers a synergistic pathway to develop cost‐effective, efficient, and sustainable adsorbents for real‐time industrial wastewater treatment. Comparative analysis shows that FBC‐CNC holds superior potential over conventional materials, especially under continuous flow conditions. However, for better clarity, a comparative table has been shown in Table S3 indicating the types and names of adsorbents and adsorbates, maximum removal %, Removal capacity, rate of adsorption, pH ranges during the experimental sessions, regeneration cycles, and references.

## 7 Mathematical Modeling and Proposed Synergistic Mechanism

### 7.1 Mathematical Models for Column Adsorption Study

Adsorption behavior is commonly analyzed using equilibrium isotherms and kinetic models to describe the interaction between pollutants and the adsorbent surface. Fixed‐bed continuous column adsorption studies, commonly applied, provide useful insights into adsorbent behavior and performance under different conditions. This approach is beneficial for selecting suitable adsorbents while ensuring environmental and economic sustainability. The process involves both external and internal mass transfer, influenced by factors such as flow rate, bed height, adsorbent surface area, porosity, particle size, adsorbate concentration, and temperature. Advanced bio‐nanocomposites, such as cellulose nanocrystals (CNC) integrated with functionalized biochar (FBC), enhance adsorption efficiency due to their high surface area, mesoporosity, and abundant functional groups, offering sustainable, biodegradable, and biocompatible alternatives to synthetic polymers [16, 31, 63] Meanwhile, various analytical techniques, including titration, chromatography, and spectrophotometry, help analyze adsorption kinetics, such as adsorption capacity, performance, breakthrough curves, and breakthrough time. Factors like flow rate, bed height, surface area, porosity, particle size, adsorbate concentration, mass transfer efficiency, and temperature significantly affect the breakthrough curve. Several adsorption models are used to predict breakthrough curves and optimize the adsorption process, providing insights into process efficiency and operating conditions [14]. Several mathematical models are used to predict breakthrough curves and optimize adsorption performance in fixed‐bed columns. These models facilitate the design, optimization, and performance evaluation of continuous adsorption systems by correlating adsorption kinetics, thermodynamics, isotherms, and mass transfer processes, thereby guiding the selection of efficient CNC‐FBC‐based nanoadsorbents for industrial wastewater treatment. These models estimate breakthrough curves (in Figure 9A) based on adsorption kinetics, thermodynamics, isotherms, and mass transfer, supporting accurate measurement of toxicant removal from effluents. However, for better clarity, they are discussed below and in Table 2.

**TABLE 2:** In‐Depth comparative analysis of fixed‐bed column adsorption models in continuous systems: evaluation of predictive capabilities, data requirements, assumptions, limitations, and industrial applications.

Adsorption model	Predictive capability	Complexity and data requirements	Assumptions	Limitations	Applications
Thomas Model	Effective for non‐linear isotherms and gives detailed breakthrough behavior	More complex	Assumes second‐order kinetics and Langmuir isotherm for adsorption, neglecting intra‐particle diffusion and internal mass transfer.	Assumes non‐ideal flow and ignores pressure and temperature variations.	Prediction of adsorption capacity and optimization/design of adsorption columns.
Yoon‐Nelson Model	More accurate for predicting the 50% breakthrough curve.	Simplest model and requires minimal data.	It assumes first‐order kinetics and a constant time to reach 50% breakthrough.	It ignores diffusion effects, lacks full insight into the process, and may not predict multi‐component system behavior accurately.	Optimize adsorption process, simulate breakthrough curves, and assess performance.
Clark Model	Predicts the initial adsorption behavior and doesn't perform accurately for complex solution.	Moderate complexity and requires minimal data.	The Clark model assumes a constant flow rate, adsorption controlled by surface interactions, no diffusion resistance, first‐order kinetics, and a single solute system in a uniformly packed bed.	Not ideal for a heterogeneous adsorption system.	This model is used to design adsorption systems, predict breakthrough times, estimate adsorption capacity, and evaluate early‐stage performance in single‐component adsorption processes.
Wolborska Model	Effective to predict initial adsorption characteristics and breakthrough curves.	Simpler and requires minimal data	Assumes the entire adsorption process in a continuous fixed‐bed column is controlled by external mass transfer resistance.	Assumes external mass transfer is the sole limiting factor and may not be suitable for high‐concentration systems.	To evaluate the breakthrough behavior at lower concentration levels when external film diffusion is the primary factor
Adam‐Bohart Model	Effective to estimates the initial adsorption phase.	Moderate complexity, emphasizing initial adsorption performance.	The process is dominated by surface reaction kinetics, ignoring mass transfer effects, with a uniformly packed bed. Free of axial dispersion	Accurate in early adsorption but may not predict long‐term performance or handle complex mixtures.	Effective for predicting initial performance, particularly suitable for new adsorbents.
BDST Model	It is ideal for predicting bed depth and service time.	Straightforward and needs minimal parameters.	Mass transfer effects are assumed to be minimal	It may overlook complexity (not suitable for all system) also ignore diffusion and resistance effects	Balancing bed depth and designing adsorption columns to optimize adsorbent performance and service life.
Modified Dosage Response Model	Suitable for predicting initial adsorption behavior	Suitable for initial performance predictions, being straightforward and requiring limited data.	Follows first‐order kinetics and assumes a linear concentration‐adsorption relationship.	It may not predict long‐term behavior and is limited to a homogeneous system.	Beneficial for optimizing adsorption performance.

#### 7.1.1 Thomas Model

The Thomas model is generally used to describe breakthrough curves, design adsorption columns, optimize performance, and evaluate adsorption efficiency in fixed‐bed systems [3]. It is based on second‐order reaction kinetics and assumes that adsorption follows the Langmuir isotherm, with negligible intra‐particle diffusion and internal mass transfer. This model offers better adsorption kinetics related to the Bed Depth Service Time (BDST) model but may introduce some complexity, especially in cases of substantial heterogeneous adsorption, internal diffusion, or non‐Langmuir behavior.

#### 7.1.2 Yoon‐Nelson Model

The Yoon‐Nelson model operates under constant flow conditions and assumes that adsorption follows first‐order kinetics. It is empirically used to optimize, model breakthrough curves, and estimate the performance of a fixed‐bed column during adsorption studies, which provides a useful empirical approach for analyzing how adsorbate concentration changes over time [188]. This model is a simple approach with few parameters, making it particularly suitable for estimating the time at which 50% breakthrough occurs.

#### 7.1.3 Clark Model

The Clark model is constructed to define the flow characteristics in continuous fixed‐bed column adsorption studies, incorporating mass transfer principles alongside the Freundlich isotherm [189]. It assumes that the adsorption rate stays constant throughout the process, representing a simple adsorption mechanism, while the adsorbent's capacity remains effective over an extended period. This model is effective for a more uniform system and not ideal for a heterogeneous adsorption system.

#### 7.1.4 Wolborska Model

The Wolborska model is designed to evaluate the breakthrough behavior at lower concentration levels, assuming that the entire adsorption process in a continuous fixed‐bed column is controlled by external mass transfer resistance. This model is particularly applicable when external film diffusion is the primary factor [9].

#### 7.1.5 Adam–Bohart Model

The Adams–Bohart model, based on second‐order kinetics, describes the adsorption process in a continuous fluid system. It assumes that the adsorption rate is proportional to both the residual capacity of the adsorbent and the concentration of the adsorbate [10]. This model is primarily used for analyzing the initial breakthrough curve rather than the full adsorption profile. It operates under the assumption that surface reaction kinetics dominate the process, neglecting mass transfer effects. Unlike simpler empirical models such as BDST, the Adams–Bohart model offers a deeper understanding of adsorption kinetics in fixed‐bed columns. However, it demands more experimental data to accurately determine its parameters.

#### 7.1.6 Bed Depth Service Time (BDST) Model

The BDST model is useful for assessing lead time and adsorption capacity in a fixed bed column prior to breakthrough, assuming uniform distribution of the adsorbent's capacity. It is based on the premise that the column exhibits a linear isotherm, no axial dispersion, and first‐order kinetics under steady flow conditions, where mass transfer effects are assumed to be minimal, and the adsorption rate is proportional to the remaining capacity of the adsorbent [3]. The BDST model is easy to apply and offers a fast estimation of breakthrough behavior, but it may not entirely reflect the fundamental adsorption mechanism.

#### 7.1.7 Modified Dosage Response Model

The Modified Dose‐Response Model (MDRM) is particularly useful for predicting breakthrough behavior in multilayer adsorption within a continuous fixed‐bed column system. This empirical model helps reduce errors associated with the Thomas model and is beneficial for optimizing adsorption conditions such as adsorbent dosage, flow rate, and bed height [96]. For better clarity, a curve fitting regarding the above‐mentioned models has been shown in Figure 9A, and the in‐depth comparative analysis of all models is shown in Table 2.

### 7.2 Proposed Synergistic Mechanism

The adsorption mechanism is governed by multiple molecular‐level interactions rather than generalized physisorption and chemisorption phenomena. The high adsorption efficiency of the developed bionanocomposites originates from their unique structural and surface characteristics, including a large specific surface area, hierarchical porosity, and the presence of abundant oxygen‐ and nitrogen‐containing functional groups such as hydroxyl (─OH), carboxyl (─COOH), and amine (─NH_2_). These functional moieties act as active binding sites and play a decisive role in governing the interaction mechanisms with different classes of pollutants. For instance, Ni^2^^+^ ions, chemisorption predominantly occurs via coordination complexation with oxygen‐ and nitrogen‐containing functional groups (─COO^−^, ─OH, ─NH_2_), where Ni^2^^+^ acts as a Lewis acid and surface ligands act as electron donors, forming inner‐sphere surface complexes such as ≡COO–Ni^+^ [1]. Additionally, ion‐exchange mechanisms may occur between protonated carboxyl groups and metal ions. In contrast, an anionic dye, Eosin Y, is primarily adsorbed through electrostatic attraction with protonated amine groups (─NH_3_^+^) under acidic conditions, along with *π*–*π* stacking interactions between the aromatic structure of the dye and the graphitic domains of biochar. Hydrogen bonding further stabilizes the adsorption complexes. The adsorption behavior is strongly pH‐dependent, where protonation of amine groups enhances dye removal, while deprotonation of carboxyl groups promotes metal ion binding. At lower pH, protonation of amine groups enhances anionic dye adsorption, whereas at higher pH, deprotonation of carboxylic groups increases the availability of negatively charged sites, favoring metal ion binding. This pH‐responsive behavior enables the selective and simultaneous removal of different contaminants. Physisorption contributes through van der Waals forces and multilayer formation, whereas chemisorption ensures strong and selective binding. Furthermore, intra‐particle diffusion facilitates the transport of pollutants into the microporous structure, increasing contact with active sites and enhancing adsorption capacity [190]. The synergistic integration of electrostatic attraction, coordination bonding, *π*–*π* interaction, and pore diffusion enables the simultaneous and efficient removal of both heavy metals and organic dyes, as schematically represented in Figure 9B.

## 8 Challenges and Future Prospects

To get effective execution in industrial sectors, numerous challenges may arise. To overcome these issues, a comprehensive and complete analysis should necessarily consider the following challenges and future prospects.

### 8.1 Challenges

Despite the promising adsorption performance of cellulose nanocrystal–biochar nanocomposites, several challenges must be addressed before their large‐scale implementation in wastewater treatment systems can be realized. One of the primary concerns is the difference between laboratory‐scale performance and real industrial conditions. Most reported studies are conducted using single‐contaminant systems under controlled environments. In contrast, industrial wastewater typically contains a complex mixture of dissolved salts, organic compounds, and suspended particles. These components can compete for active adsorption sites, leading to reduced removal efficiency. The presence of high ionic strength in wastewater may also influence electrostatic interactions between the adsorbent surface and pollutant species. Variations in pH can alter the surface charge of the material as well as the speciation of contaminants, further affecting adsorption behavior. In addition, suspended solids and macromolecular organic matter can accumulate on the adsorbent surface, causing pore blockage and limiting mass transfer. Such fouling phenomena may significantly reduce the long‐term performance of the adsorbent. Another critical challenge is the scalability of material synthesis [191, 192, 193, 194, 195, 196]. The production of cellulose nanocrystals often involves chemical treatments and purification steps that may increase cost and complexity when scaled up. Similarly, the activation and functionalization of biochar require careful control of processing conditions, which may pose difficulties in large‐scale operations. Regeneration and reuse of the adsorbent also remain important considerations. Repeated adsorption–desorption cycles may lead to structural degradation or loss of active functional groups, thereby reducing adsorption efficiency over time. Furthermore, the integration of these materials into continuous treatment systems, such as fixed‐bed adsorption columns, introduces additional engineering challenges. These include pressure drop, flow distribution, and long‐term operational stability [197]. Although the entire process may encounter a few challenges, these challenges can be reduced or mitigated substantially by following optimum parameters and monitoring the process over time by changing concentration and system conditions. In addition, a highly porous and functional bio‐nanocomposite, which showed better morphological structure, may perform better compared to others. While this technique can be helpful for reducing operational costs, the hindrances or process difficulties cannot be neglected.

### 8.2 Future Prospects

#### 8.2.1 Development of Green Functionalization and Cost‐Effective Methods

Explore waste‐derived CNC sources and apply eco‐friendly enzymes, modifiers, and chemicals to discover cost‐effective methods that help establish a green environment.

#### 8.2.2 Developed Regeneration and Materials Sustainability Strategies

Enhance structural stability for prolonged application and use chemical‐free regeneration and electrochemical oxidation techniques in fixed‐bed column adsorption.

#### 8.2.3 Implement a Multifunctional Adsorption System

Combine nanocomposites with other treatments, such as biological treatment, advanced oxidation, or ultrafiltration, and develop catalysts for the catalytic degradation of contaminants.

#### 8.2.4 Practical Industrial Applications and AI‐Driven Optimization

Conduct economic feasibility studies and pilot‐scale projects in textiles, heavy metal treatment, and pharmaceuticals for commercial adoption. Additionally, utilize AI tools to enhance breakthrough curve prediction and optimize adsorption processes.

#### 8.2.5 Incorporating Sustainability and Circular Economy Principles

Enhance the use of industrial and agricultural waste for the production of biochar and biopolysaccharide, while promoting resource recovery through the implementation of closed‐loop effluent treatment. By exploring emerging research opportunities and addressing these prospects, CNC‐loaded functionalized biochar‐based nanoadsorbents can become a sustainable and effective solution for industrial wastewater treatment.

## 9 Conclusions

Production of multifunctional biopolymeric nanocomposites/nanosorbents from naturally occurring plant waste biomass‐derived lignocellulosic fibers, biochar, activated carbon, etc., by developing a cost‐effective and environmentally friendly technology for the selective separation of hazardous toxic pollutants from bulky industrial wastewater could be a new challenge to the researchers. To mitigate this issue, various beneficial methods of extraction and modification of the considered precursors, such as cellulose, CNC, biochar, and functionalized biochar, have been comprehensively reviewed in this current study. Moreover, several state‐of‐the‐art fabrication techniques (like solution casting, spin coating, hot press, dip coating, melt intercalation, in situ polymerization, sol–gel, electrospinning, phase inversion, selective laser sintering, etc.) have also been proposed and discussed for effective manufacturing of CNC‐FBC‐based biopolymeric nanoadsorbents by using those previously modified precursors. Additionally, a considerable number of advanced characterization techniques have been discussed, indicating their scope, contribution, sampling techniques, working principle, operational conditions, major findings, real‐time examples, including data analysis of similar materials and composites for better clarity. Besides these, the particular mode of application of the newly produced CNC‐FBC‐based biopolymeric nanoadsorbents in industrial wastewater purification technology, such as a fixed‐bed continuous column adsorption system, has been conferred effusively for better selectivity. It is important to mention that the considered CNC‐FBC‐based biopolymeric nanoadsorbents could be a much more promising candidate in the bulky wastewater purification technology as an alternative to the hazardous fossil‐based composite materials due to numerous outstanding properties. Such as cost effectiveness, greater availability, higher performances, eco‐friendly nature, relatively higher specific surface area, exceptional physicochemical, thermomechanical, microstructural, and morphological properties, which must boost their adsorption capacities (around 99%) against various toxic pollutants if CNCs were effectively incorporated onto the functionalized biochar during the production and processing steps. This review also proposed a synergistic adsorption mechanism offering an actual reason and pathway of the selective separation of toxic pollutants from the bulky wastewater bodies by providing the insights of the chemisorption due to the chemical bonding like covalent/co‐ordination among the active sites of CNC‐AC based bionanosorbents and the toxicants, both mono‐layer and multi‐layer physisorption as a result of the Vander Waals forces, dipole momentum, and hydrogen bonding (usually occurred on the active receptor sites and porous microstructure/mesoporous surface peripheral structure) along with interparticular diffusion and deep penetration due to the presence of pour tube/pour canal in the morphostructure of the applied bionanoadsorbents. And lastly, some widely used, well‐organized mathematical models have also been suggested along with sufficient discussions and scientific hypotheses that could be beneficially applied to predict and evaluate the obtained experimental adsorption data/BTC curve for a better understanding of the definite sorption behavior of the considered CNC‐FBC biopolymeric nanosorbents against the particular toxic pollutants.

## Author Contributions

**Md. Mahmudur Rahman**: conceptualization, fund acquisition, methodology, software, validation, formal analysis, data curation, writing – review and editing. **M Mohinur Rahman Rabby**: writing – review and editing. **G.M Musfiq Ismam**: software, validation, formal analysis. **Salah Knani**: fund acquisition. **Md. Khalid Al Zuhanee**: writing – original draft. **Reem Alreshidi**: visualization. **Faisal Ahmed Naiem**: formal analysis, visualization.

## Funding

The authors extend their appreciation to the Deanship of Scientific Research at Northern Border University, Arar, KSA for funding this research work through the project number ″NBU‐FFR‐2026‐3547‐01. As well as to the Ministry of Science and Technology (MOST), the People's Republic of Bangladesh, and the Bangladesh Council of Scientific and Industrial Research (BCSIR), for their joint funding to conduct this current work through the Research and Development Project under grant numbers (G.O.39.02.0000.011.14.200.2025.1326 and G.O.39.00.0000.207.0004.25.174).

## Conflicts of Interest

The authors declare no conflicts of interest.

## Supporting information




**Supporting File**: gch270107‐sup‐0001‐SuppMat.docx.

## Data Availability

The data that support the findings of this study are available from the corresponding author upon reasonable request.
