# Supplementary material for: Recent Advances in the Fabrication of Structural Nanocellulose‐Loaded Functionalized Biochar‐Based Biopolymeric Nanocomposites for Industrial Wastewater Treatment by Continuous Adsorption With Modeling: A Cutting‐Edge Review
Source: Glob Chall. 2026 Apr 24;10(4):e70107. doi: 10.1002/gch2.70107 (PMC13107170; doi:10.1002/gch2.70107)
Supplement: Supplementary file 1 — Supporting File: gch270107‐sup‐0001‐SuppMat.docx. [file GCH2-10-e70107-s001.docx]

**Supplementary information**

**Recent advances in the fabrication of structural nanocellulose-loaded functionalized biochar-based biopolymeric nanocomposites for industrial wastewater treatment by continuous adsorption with modeling: A cutting-edge review**

Md. Mahmudur Rahman^a*^; M Mohinur Rahman Rabby^d^; G.M Musfiq Ismam^e^; Md. Khalid Al Zuhanee^d^; Salah Knani^c^; Reem Alreshidi^f^; Faisal Ahmed Naiem^e^

^a^ BCSIR, Rajshahi Laboratory, Bangladesh Council of Scientific and Industrial Research (BCSIR), Rajshahi-6206, Bangladesh.

^c^ Center for Scientific Research and Entrepreneurship, Northern Border University, 73213 Arar, Saudi Arabia.

^d^ Department of Chemical Engineering, Rajshahi University of Engineering and Technology (RUET), Rajshahi-6204, Bangladesh.

^e^ Department of Mechanical Engineering, Rajshahi University of Engineering and Technology (RUET), Rajshahi-6204, Bangladesh.

^f^ Department of Physics, College of Science, Northern Border University, 73213 Arar, Saudi Arabia.

^*^Correspondence to: Md. Mahmudur Rahman (E-mail: [shamrat.acce@gmail.com](mailto:shamrat.acce@gmail.com))

**Table S1.** Accountable active binding sites including their specific wavenumber, nature, and feature of peaks which could have usually been presented in the structure of the considered biopolymeric samples as per FTIR-ATR analysis [5,12,30,45,61,62] .

| **Particular peaks (cm^− 1^)** | **Intensity type** | **Responsible group** |
| --- | --- | --- |
| 3700–3400 | Broad & Stretching | –OH of α-cellulose |
| 3400–3200 | Sharp & stretching | N–H/ −NH2 of Amine |
| 3500 | Sharp & stretching | N–H from Amide |
| 3100–3000 | Sharp & stretching | −C==C– |
| 2960–2840 | Sharp & stretching | C–H |
| 2590–2540 | Broad & stretching | S–H |
| 1760–1710 | Sharp & stretching | –C==O of hemicelluloses |
| 1685–1655 | stretching | α, β – unsaturation |
| 1690–1640 | Sharp & stretching | –N==O |
| 1600-1560 | stretching | C==O (Amide) |
| 1600–1450 | Sharp & stretching | C==C |
| 1480–1400 | Symmetric bending | CH2 |
| 1567,1380 | stretching | NO2 |
| Above 1500 | Broad & stretching | C==O, –NH, C==C, C==N |
| Below 1500 | Bending vibrations | C–C |
| 1365–1400 | Bending | –CH (deformation) |
| 1360-1260 | stretching | –C–O |
| 1150–1010 | Sharp & stretching | –C–O–C |
| 1050 - 1020 | Symmetric stretching | C–OH of lignin |
| 850–640 | stretching | –NO |
| 770–635 | Bending | –C–C– deformation |
| 550–420 | stretching | S–S deformation |
| Below 500 | Bending & stretching | Si–O, Mn–O, Ni–O, etc. |


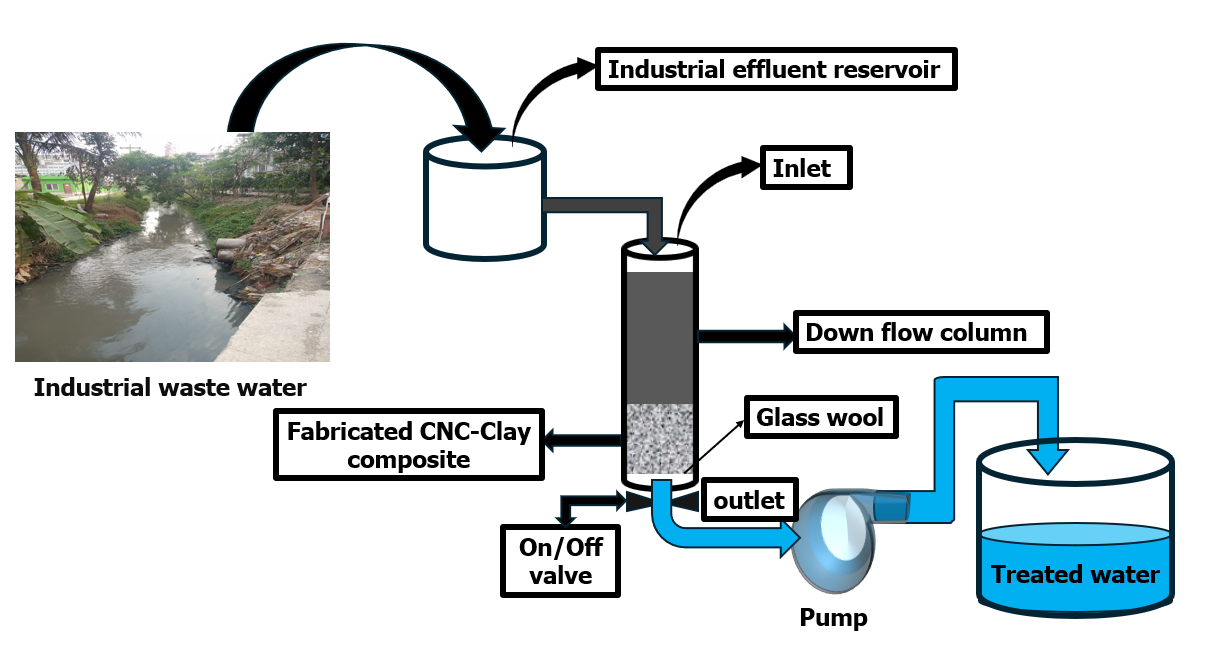


**Fig. S1.** Sketch diagram addressing the experimental setup of a fixed-bed downflow column continuous adsorption study in lab scale/piloting for the industrial wastewater treatment by using CNC-FBC biopolymeric nanoadsorbents.

**Table S2:** Summary of the different desorption processes according to the different pollutants adsorbed on various biopolymeric nanoadsorbent during the experimental sessions.

| **Adsorbent** | **Reagent** | **Contaminant** | **Cycles** | **Efficiency (%)** | **Reference** |
| --- | --- | --- | --- | --- | --- |
| LaNiO₃/g-C₃N₄ nanocomposite | Natural Sunlight | Reactive Black 5, Methylene Blue, Tetracycline hydrochloride | 4 | 94,  98.6,  88.1 consecutively | [176] |
| CeO₂/ZnO nanocomposite | UV light | Methylene Blue | 4 | 74.10 | [177] |
| Methylene BlueFe₃O₄/cyclodextrin polymer nanocomposite | Desorption agents: 0.01 M HNO₃ (96% Pb²⁺ recovery), 0.1 M Na₂EDTA (94.2% Pb²⁺ recovery), 0.02 M H₃PO₄ (82.7% Ni²⁺ recovery) | Pb²⁺,  Cd²⁺,  Ni² | 4 | 99.9 | [178] |
| Magnetite nanoparticles (Fe₃O₄) | Sodium hydroxide | Pb²⁺ | 4 | N/A | [179] |
| CNF | 0.5 M HNO_3_ | Cd^2+^,  Pb^2+^,  Ni^2+^ | 4 | 99.2- Cd^2+^  96.8- Pb^2+^  90.6- Ni^2+^ | [180] |
| CNC-Pectin Composite | 0.2 N HCl | Uranium (VI) | 4 | >98 | [1] |
| AC-MC nanocomposites | 0.2 M NaOH | CV,  Pb^2+^ | 5 | 83.13  79.52 | [11] |
| ZIF-8/CNC nanohybrid | N/A | Cd (II) | 5 | 93.80 | [181] |
| CNC-MC nanocomposites | 0.2 M NaOH | Ni^2+^  CR | 3 | 67.95  76.65 | [12] |

**Table S3:** A comparative data indicating the types and names of adsorbents and adsorbates, maximum removal %, Removal capacity, rate of adsorption, pH ranges during the experimental sessions, regeneration cycles and references addressing industrial wastewater treatment by applying various biopolymeric nanoadsorbents.

| **Adsorbents** | **Adsorbate** | **Removal (%)** | **Adsorption Rate (min)** | **q_max_ (mg/g)** | **Range of P^H^** | **Regeneration Cycles** | **Reference** |
| --- | --- | --- | --- | --- | --- | --- | --- |
| Bentonite-Clay/CNT | Dyes | 89.9 | 120 | 550 | 3 | - | [182] |
| ZIF-8/CNC nanohybrid | Cd(II) | 93.8 | 120 min | 423 | 7 | 5 | [181] |
| Novel Fe_3_O_4_/hydroxyapatite/β-cyclodextrin nanocomposites | Cd^2+^  and  Cu^2+^ |  | 60 | 100.0  and  66.66 | 6 | 5 | [183] |
| CNC-AC biopolymeric Nanocomposites | Pb^2+^  CR | 85.01  81.33 | N/A | 538.91  and 455.70 | 4.2 | 5 | [14] |
| Fe(III)-imprinted polymer (IIP) with acrylic acid/EGDMA | Fe(III) ions in Cr(III)-containing solution | 94.43 | 900 | 114.25 | 3-3.5 | - | [184] |
| Chitosan/PEO/Activated Carbon Nanofibrous Membrane | Cr(VI) | 97.6 | 11 | 261.1 | 3 | - | [185] |
| Crystalline nanocellulose-Modified coal biocomposites | Ni2+ and Congo red | 67.95  76.65 | N/A | 328.7  and  478.3 | 4.9 | 3 | [12] |
